# Toxicity Study of 28-Day Subcutaneous Injection of Arctigenin in Beagle Dogs

**DOI:** 10.3389/fphar.2019.01218

**Published:** 2019-10-16

**Authors:** Jie Li, Yun-gang Lv, Li-hong Pan, Fang-fang Yao, Tao Peng, Yu-jun Tan, Gui-Min Zhang, Zhong Liu, Jing-chun Yao, Yu-shan Ren

**Affiliations:** ^1^National Engineering Laboratory of High Level Expression in Mammalian Cells, Lunan Pharmaceutical Group Co. Ltd., Linyi, China; ^2^State Key Laboratory of Generic Manufacture Technology of Chinese Traditional Medicine, Lunan Pharmaceutical Group Co. Ltd., Linyi, China; ^3^Center for New Drug Safety Evaluation of Lunan Pharmaceutical, Lunan Pharmaceutical Group Co. Ltd., Linyi, China; ^4^Department of Radiology, Shenzhen University General Hospital, Shenzhen University Clinical Medical Academy, Shenzhen, China; ^5^National Engineering and Technology Research Center of Chirality Pharmaceutical, Lunan Pharmaceutical Group Co. Ltd., Linyi, China

**Keywords:** arctigenin, toxicity, toxicokinetics, NOAEL, inflammation

## Abstract

Our previous studies have investigated the systematic pharmacokinetic characteristics, biological activities, and toxicity of arctigenin. In this research, the potential toxicities of arctigenin in beagle dogs were investigated *via* repeated 28-day subcutaneous injections. Beagle dogs were randomly divided into control, vehicle [polyethylene glycol (PEG)], and arctigenin 6, 20, 60 mg/kg treated groups. The whole experimental period lasted 77 days, including adaptive period (35 days), drug exposure period (animals were treated with saline, PEG, or arctigenin for 28 consecutive days), and recovery period (14 days). Arctigenin injection (60 mg/kg) affected the lymphatic hematopoietic, digestive, urinary, and cardiovascular systems, and all the impact on these tissues resulted in death in five dogs (three female and two male dogs); 20 mg/kg arctigenin injection resulted in toxic reactions of the lymphatic hematopoietic and digestive systems; and 6 mg/kg arctigenin and PEG injection did not lead to significant toxic reactions. Meanwhile, there were no sexual differences of drug exposure and accumulation when dogs underwent different dosages. As stated previously, the toxic target organs of arctigenin administration include lymphatic hematopoietic, digestive (liver and gallbladder), urinary (kidney), and cardiovascular (heart) systems, and the no observed adverse effect level (NOAEL) of arctigenin is less than 6 mg/kg.

## Introduction

Greater burdock (*Arctium lappa*) is widely known as niubang in China, and was introduced into Japan and Korea, and widely eaten in the region (Baba et al., 2018; Lee et al., 2018). During the Middle Ages, it was used in Europe as a vegetable, and even now, it is still used in Italy, Brazil, and Portugal (Miguel et al., 2010; Meinhart et al., 2017). In addition, the root of burdock was traditionally used in Britain as a flavoring in the herbal drink made with dandelion and burdock, and is still commercially produced (Watkins et al., 2012). In the 20^th^ century, burdock was advocated in culinary use due to the increasing popularity of the macrobiotic diet.

Arctigenin, the aglycone of arctiin, was found in greater burdock and was reported to have antiviral (Chen et al., 2016; Dias et al., 2017) and anticancer (Jiang et al., 2015; Feng et al., 2017) effects *in vitro*. In a mouse model of Japanese encephalitis, arctigenin showed effective function (Swarup et al., 2008). Further, arctigenin was reported to act as an agonist of adiponectin receptor 1 (AdipoR1) (Sun et al., 2013). Our previous study investigated the systematic pharmacokinetics of arctigenin in rats and beagle dogs (Li et al., 2017). The results showed that compared with oral administration, subcutaneous injection of arctigenin exhibited a strong absorption capacity in beagle dogs (absorption rate < 1 h), a high absorption degree (absolute bioavailability > 100%), and a strong elimination ability (t_1/2_ < 2 h). Subsequently, another study investigated the efficacy and potential mechanism of arctigenin on liver cancer (Sun et al., 2018). Our results have shown that arctigenin significantly inhibited cell growth, migration, invasion, and colony formation of HepG2 cells *via* reduction of the gankyrin expression in both mRNA and protein.

Although multiple bioactivities of arctigenin were found *in vivo* and *in vitro* (Wu et al., 2014; Liu et al., 2015; Su et al., 2015; Huang et al., 2017), the systemic evaluation of this chemical’s toxicology was rarely reported. In the present research, the potential toxicities of arctigenin in beagle dogs were investigated *via* repeated 28-day subcutaneous injection. The characteristics, degrees, dose effects, time effects, and reversibility of toxic reactions and the target organs or tissues after arctigenin exposure were investigated.

## Materials and Methods

### Chemicals and Agents

**Figure 1:**
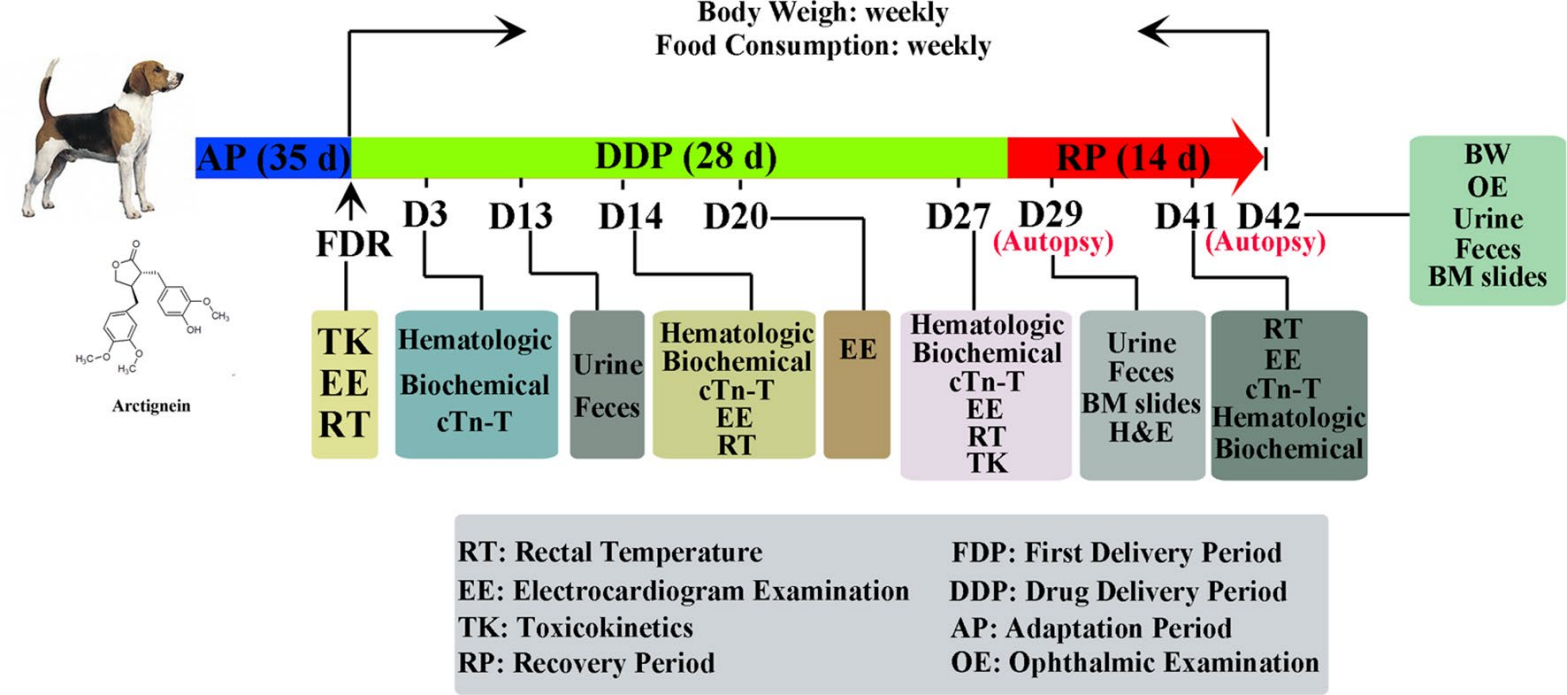
Representative experimental scheme.

Arctigenin injection (purity was 99.8%, 500 mg/10 ml, Cat. #120801) and polyethylene glycol (PEG) solution (80%, Cat. #120804) were supplied by Shandong New Time Pharmaceutical Co. (Linyi, Shan Dong province, China). The chemical structure of arctigenin is shown in **Figure 1**. The saline (0.9% NaCl, Cat. #M12101307) was purchased from Kelun Pharmaceutical Co. (Chengdu, Sichuan Province, China). Pentobarbital sodium was from Sigma-Aldrich (Cat. #93ET2904K, St. Louis, MO, USA).

### Test Article Solutions Analysis

Before the first drug exposure, the relative errors (RE) between measured concentrations and theoretical value of fresh solutions of arctigenin (6 mg/kg, 20 mg/kg, and 60 mg/kg) were 13.2%, 10.8%, and 7.5%, respectively. The upper, middle, and lower solution’s relative standard deviations (RSD) were 3.7%, 0.5%, and 1.7%, respectively.

On the day of drug exposure, the RE between measured concentrations and theoretical value of first fresh solutions of arctigenin (6 mg/kg, 20 mg/kg, and 60 mg/kg) were −3.9%, −3.0%, and −1.9%, respectively. The upper, middle, and lower solution’s RSDs were 2.8%, 1.2%, and 3.3%, respectively.

On the day of last drug exposure, the RE between measured concentrations and the oretical value of first fresh solutions of arctigenin (6 mg/kg, 20 mg/kg, and 60 mg/kg) were 9.7%, 15.2%, 14.1%, respectively. The upper, middle, and lower solution’s RSDs were 0.4%, 1.7%, and 1.1%, respectively.

These data demonstrated that the concentrations and uniformities complied with the acceptable standard of the scheme (data not shown).

### Animals, Grouping, and Drug Administration

Representative experimental scheme is shown in **Figure 1**. In brief, 40 beagle dogs (20 male/20 female), purchased from Guangzhou General Pharmaceutical Research Institute Co. (Guangzhou, Guangdong Province, China), were randomly divided into five groups, as follows: (A) a control group (animal no. 1F01-1F04 were female, 1M01-1M04 were male), (B) a PEG-treated group (animal no. 2F01-2F04 were female, 2M01-2M04 were male), (C) an arctigenin 6 mg/kg group (5 mg/ml, diluted by PEG; animal no. 3F01-3F04 were female, 3M01-3M04 were male), (D) an arctigenin 20 mg/kg group (16.7 mg/ml, diluted by PEG; animal no. 4F01-4F04 were female, 4M01-4M04 were male), and (E) an arctigenin 60 mg/kg group (50 mg/ml, diluted by PEG; animal no., 5F01-5F04 were female, 5M01-5M04 were male). The animals were fed in GLP (Good Laboratory Practice) center, which accepted by the China Food and Drug Administration (CFDA), and supplied with standard rodent diet (SPF grade, supplied by Beijing Keao Xieli Feed Co. Ltd., Beijing, China) and water ad libitum, acclimatized to a controlled temperature (23 ± 2°C), and maintained under a 12/12-h light/dark cycle. The cage beddings and water bottles were cleaned on a daily basis. The animals were allowed 2 weeks of acclimatization before the commencement of experimental procedures. The standard of feeding environment is based on People’s Republic of Chinanational standard (GB14925-2010), and the feeding environment control system is SIMATIC S7-300 automatic control system. During the time of drug administration, the dogs averaged 6–7 months of age, and body weight ranged from 5.1 kg to 6.2 kg. All animals received daily subcutaneous injections at the volume of 1.2 ml/kg; in groups A and B, saline and PEG were used in the injection, respectively. Groups C, D, and E were injected with arctigenin at different concentrations. The study was conducted in accordance with the Basic & Clinical Pharmacology & Toxicology policy for experimental and clinical studies (Tveden-Nyborg et al., 2018). These dogs have a regular body checkup everyday during experiment. All dogs in each group were anesthetized with pentobarbital sodium (Sigma, Louis, MO, USA) and euthanized at 24 h after their last dose.

### General Observation

Cage-side examinations for apparent signs (behavior, mental status, gland secretion, respiration status, feces characters, genitals, and death) of toxicity or injury were conducted once a day after daily drug exposure, and once experimental animals develop toxic symptoms, observation frequency was increased. During the recovery period, the observation was performed once a day. The standard of general observation was in accordance with the Guide for the Care and Use of Laboratory Animals published by the National Academies Press (Ed 8).

### Body Weight and Food Consumption

The body weight in all groups was measured once before the first drug exposure and then measured twice per week (on Monday and Thursday) during the drug exposure period and then measured weekly (on Thursday) during the recovery period. Food consumption was measured on one day each week over a 24-h period. On the days of the food consumption assays, 300 g animal feed was delivered, and the remaining portions were measured at the same time on the second day (on Monday), and the food consumption was calculated.

### Rectal Temperature

The rectal temperatures of all animals were recorded before drug administration and at 1, 4, and 24 h after the first drug exposure. On experiment days 14 and 27, temperatures were recorded 1 h after drug injection. Rectal temperatures were measured using a TH-212 digital thermo detector (Cat. #TH-212, Beijing high-tech Co. Beijing, China).

### Electrocardiogram (Ii Lead)

Electrocardiograms (II lead) were performed using a four-channel polygraph (Cat. #RM6240, Chengdu Instrument Factory, Chengdu, Sichuan province, China) before and at 1, 4, and 24 h after the first drug exposure; before and at 1 h after drug exposure at day 14; before and at 1 and 2 h after drug exposure on day 20; and before and at 1 h after drug exposure on day 27.

### Ophthalmic Examination

At the end of drug exposure (day 27) and recovery (day 42), tropicamide was applied to the mydriasis, cornea, iris, crystalline lens, anterior/atria chamber, and fundus oculi of all animals, and they were examined using the Binocular Indirect Ophthalmoscope (Cat. #YZ25A, 6-6 VISION TECH Co., Ltd., Suzhou, Jiangsu province, China).

### Hematologic, Biochemical, and Cardiac Troponin (ctn-T) Examination

Before drug exposure on days 3, 14, and 27, the forelimb vein blood (4.9 ml) was collected from all animals, who fasted for 12 h before sampling.

For hematological cytology assay, 1 ml of blood was anticoagulated by ethylenediamine tetraacetic acid (EDTA). For detecting clotting time, 0.9 ml of blood was anticoagulated by sodium citrate, 1800*g* for 10 min at room temperature (RT), and the plasma was separated for examination. Hematological parameters including red blood cells (RBC), hemoglobin (HGB), hematocrit (HCT), mean corpuscular volume (MCV), mean corpuscular hemoglobin (MCH), mean corpuscular hemoglobin concentration (MCHC), reticulocyte (RET%), white blood cells (WBC), neutrophils (NEU), lymphocytes (LYM), monocytes (MONO), eosinophils (EOS), basophils (BASO), platelets (PLT), prothrombin time (PT), and activated partial thromboplastin time (APTT) were detected by the Automatic Five Classification Blood Analyzer (ADVIA2120, SIEMENS, Germany) and the Automatic Hemagglutination Analyzer (CA-7000, SYSMEX, Japan), respectively.

For biochemical examination, 3 ml of blood was centrifuged (1800*g*, 10 min, RT), and serum was prepared for examination. Parameters including aspartate aminotransferase (AST), alanine aminotransferase (ALT), creatine phosphokinase (CPK), myocardial bound creatine kinase (CK-MB), alkaline phosphatase (ALP), L-lactate dehydrogenase (LDH), γ-glutamyl transpeptidase (GGT), urea (urea), troponin (TP), albumin (ALB), albumin/globulin (A/G), glucose (GLU), total bilirubin (TBIL), creatinine (crea), cholesterol (CHOL), triglyceride (TG), amylopsin (AMY-P), lipidase (LIPC), calcium (Ca), P, sodium (Na^+^), potassium (K^+^), and chloride ion (Cl^−^) were detected by Automatic Blood Biochemical Analyzer (Cobas C501, ROCHE, Switzerland).

For cardiac troponin (cTn-T) examination, 1 ml of blood was centrifuged (1800*g*, 10 min, RT), and serum was prepared for examination.

### Urine and Feces Examination

Urine and feces examinations were conducted once on days 13 and 29 and at the end of the recovery period. Urine parameters including glucose (GLU), bilirubin (BIL), ketones (KET), specific gravity (SG), occult blood (BLO), pH, protein (PRO), urobilinogen (URO), nitrogen (NIT), and WBC were detected by Urine Analyzer (CLINITEK STATUS, BAYER, Germany).

### Bone Marrow Slides Examination

Bone marrow slide examinations were conducted once on day 29 and at the end of the recovery period. The beagle dogs were anesthetized by pentobarbital sodium (30 mg/kg intravenous), bone marrow was stained by Wright-Giemsa, and parameters including granulocyte series, erythrocyte series, lymphocyte series, monocyte series, megakaryocyte, and karyocyte were evaluated.

### Toxicokinetics Study

From 0 min to 8 h after arctigenin administration, 1 ml of the dogs’ forelimb vein blood was obtained. The blood samples were centrifuged at 3000 rpm for 10 min at 4°C to obtain the plasma (45 μl). After adding 5 μl internal standard (IS) and 145 μl methanol, centrifuging at 10,000 rpm for 10 min at 4°C, and repeating this step once to obtain the supernatant, 100-μl samples were collected, and arctigenin concentrations were detected by HPLC-MS/MS. Kinetic parameters (AUC_0-t_, C_max_, and T_max_) were calculated.

### Autopsy and Histopathology Examination

The autopsy was conducted at the end of the drug exposure (on day 29, four dogs per group were autopsied) and at the recovery period (the remaining dogs: four in control, PEG, and arctigenin 6 mg/kg groups; four in arctigenin 20 mg/kg group; and one in arctigenin 60 mg/kg group). The brain, spleen, thymus, lung, heart, liver, testis (bilateral)/ovaries (bilateral), kidneys (bilateral), and adrenal glands (bilateral) were separated and weighed.

To detect tissue injury, the histopathology examination was performed as previously described (Akindele et al., 2014). In brief, dog tissues were fixed in 4% paraformaldehyde buffer for 24 h, dehydrated in graded alcohol (70, 90, 95, and 100%), and embedded in paraffin. Subsequently, paraffin blocks were cut into 2-µm sections and then subjected to routine hematoxylin-eosin (H&E) staining according to previous protocols (Fei et al., 2017). The photomicroscopic assessment using BX53 photomicroscope (Olympus, Tokyo, Japan) and the histopathology slides were viewed at various magnifications (×40, ×100, and × 400) to detect pathological lesions.

### Statistical Analysis

Data were calculated and analyzed with Excel 2010 (Microsoft, Redmond, WA, USA) and SPSS V19.0 (SPSS, Inc., Chicago, IL, USA). Toxicokinetic parameters, including C_max_, T_max_, and area under the curve (AUC), and the compartment model were analyzed by the Drug and Statistics (DAS) 3.0 software (Chinese Mathematical Pharmacology Society, Beijing, China). All values were presented as mean ± standard deviation (S.D.) and calculated using an unpaired *t* test by SPSS Statistics V19.0.

## Results

### Death/Articulo Mortis of Animals

At the beginning of the drug exposure period, one animal in the arctigenin 60 mg/kg group (female, NO. 5F04) had onset of symptoms, including loose stools, pronation, and feeding reduction. After seven consecutive days of drug exposure, convulsion, respiratory slow down, deeper breathing, and limb rigidity, and, subsequently, death were observed in this animal. Four animals in the arctigenin 60 mg/kg group (two females and two males, NO. 5M04, 5F02, 5F03, and 5M01) showed activity and feeding reductions after the first drug exposure, and from day 8 to day 14 of drug exposure period. All these animals presented pronation, myasthenia of limbs, magersucht, yellow skin, dark red diarrhea, respiratory slow down, deeper breathing, and articulo mortis on days 20, 22, 23, and 26, respectively. As shown in **Table 1**, four animals in articulo mortis presented hemopoiesis inhibition (erythron index reduction, reticulocyte ratio increase), abnormal liver function index (ALP, ALT, AST, LDH, TBIL), electrolyte disturbances (K^+^, Cl^−^), clotting time prolongation (APTT, PT), and abnormal kidney function (crea, urea, urine glucose, occult blood, urobilinogen) (**Table 2**).

**Table 1:** The urine, viscera weight, and electrocardiogram parameters of articulo mortis animals.

Urine examination					Viscera weight					Electrocardiogram examination				
PAR	5F02	5F03	5M01	5M04	PAR (g)	5F02	5F03	5M01	5M04		5F02	5F03	5M01	5M04
**GLU (mM)**	5.5	5.5	5.5	5.5	**B.W (kg)**	7.7	6.65	5.8	6.55	**P-wave (ms)**	90	56	54	42
**BIL**	High	High	High	High	**Liver**	317.73	344.21	208.86	234.33	**P-R** **_inter_** **(mV)**	272	92	112	96
**KET (mM)**	TQ	1.5	–	TQ	**Spleen**	22.15	55.54	22.09	18.78					
**S.G**	1.015	1.02	1.015	<1.005	**Kidney**	72.19	68.63	57.03	69.93	**QRS-wave (ms)**	268	84	76	78
**Ery (Ery/µl)**	200	200	200	200	**Adrenal**	1.71	1.35	1.18	1.03					
**PH**	6	6	6.5	5.5	**Ovary/ Epididymis**	0.1	0.67	1.97	6.04	**Q-T** **_inter_** **(ms)**	576	274	278	266
**PRO (g/L)**	>3	1	0.3	1	**Uterus/ Testis**	0.89	1.87	0.74	2.11	**R-R** **_inter_** **(ms)**	840	750	559	630
**URO (µM)**	3.2	16	3.2	16	**Thymus**	2.26	2.51	3.98	1.54					
**NIT**	–	–	–	–	**Heart**	83.3	73.58	48.71	73.09	**HR (/min)**	71.38	80.35	107.38	95.11
**LEU (Leu/µl)**	–	500	–	500	**Lung**	81.2	132.97	65.76	77.85					
**Color**					**Brain**	67.27	79.99	71.67	75.78	**C QT** **_inter_** **(ms)**	610	302	338	310
**Clarity**														

PAR, parameter; TQ, trace quantity; GLU, glucose; KET, ketones; PRO, protein; BIL, bilirubin; URO, urobilinogen; NIT, nitrite; S.G, specific gravity; LEU, leucocytes; B.W, body weight; P-R_inter_, P-R intermittent; HR, heart rate; C Q-T_inter_, Correction q-t interphase

**Table 2:** The hematologic and biochemical parameters of articulo mortis animals.

Hematologic examination						Biochemical examination					
NO.		5F02	5F03	5M01	5M04	NO.		5F02	5F03	5M01	5M04
Gender		F	F	M	M	Gender		F	F	M	M
WBC	10^9^/L	21.24	10.62	8.97	28.26	ALP	U/L	/	990.6	1749.5	2274.1
NEU	10^9^/L	6.71	0.68	4.56	21.32	ALT	U/L	66.7	296.8	299.4	1522.5
LYM	10^9^/L	8.96	6.98	3.48	4.48	AST	U/L	66.4	392.1	254.8	1078
MONO	10^9^/L	4.37	2.3	0.76	1.93	GGT	U/L	/	-4.7	4.9	7.8
EOS	10^9^/L	0.12	0.09	0.08	0.38	CK	U/L	/	1083	558	777
BASO	10^9^/L	0.2	0.11	0.03	0.08	CK-MB	U/L	/	742	814	/
N	%	31.6	6.4	50.8	75.5	LDH	U/L	/	531	222	689
L	%	42.2	65.7	38.8	15.8	Urea	mM	2.6	30	24	3.9
M	%	20.6	21.7	8.5	6.8	Crea	µM	11.4	185.1	61.2	37
E	%	0.6	0.8	0.9	1.3	TP	g/L	/	24	28.9	50.8
B	%	0.9	1.1	0.3	0.3	ALB	g/L	/	11.5	11.8	23.9
RBC	10^12^/L	6.64	3.07	4.03	6.46	A/G	/	/	0.92	0.69	0.89
HGB	g/L	142	70	83	139	GLU	mM	/	0.1	6.69	5.79
HCT	%	45.5	22.7	27.7	42.6	TBIL	µM	/	244	188	113
MCV	fl	68.5	74.2	68.9	65.9	CHOL	mM	/	0.44	2.43	1.29
MCH	pg	21.5	22.8	20.6	21.5	TG	mM	/	1.34	1.46	1.22
MCHC	g/L	313	308	300	326	K^+^	mM	/	14.8	3.45	3.8
PLT	10^9^/L	163	57	402	608	Na+	mM	/	130	130	143
RET%	%	3.44	4.63	9.78	2.34	Cl^-^	mM	/	88	94.2	103.2
PT	s	124.3	62.4	19.3	300	Ca	mM	/	3.1	2.6	2.3
APTT	s	68.6	177.9	36.2	13.8	AMY-P	U/L	/	6.95	2.44	2.14
						LIPC	U/L	/	591	112	64.6

The electrocardiogram examination demonstrated that the animals in articulo mortis showed sinus arrhythmia, a reduced heart rate, P-wave and Q-wave disappearance, S-T and T wave elevation, and Q-T interval prolongation (**Table 1**, **Figure S1**). All these results implied that myocardial damage obstruction may have occurred in these animals.

The histopathological results showed test article subcutaneous deposition at the injection site, mammary gland, and subaxillary lymph node, which resulted in moderate-severe inflammation and foreign body reaction, including edema, hemorrhage, necrosis, and inflammatory cell infiltration. In the lymphatic hematopoietic system, subaxillary lymph node lesions appeared with mild-moderate red cell increase/phagocytosis (two of three dogs) and mild pigmentation in the lymphoid sinus (one of three dogs). In the spleen, one dog (1/5) had moderate extramedullary hematopoiesis (EMH); mild-moderate foam-like macrophage aggregation was observed in three dogs (3/5); and mild white pulp atrophy was observed in two dogs (2/5). In the thymus, two dogs (2/3) showed marked-severe atrophy.

**Figure 2:**
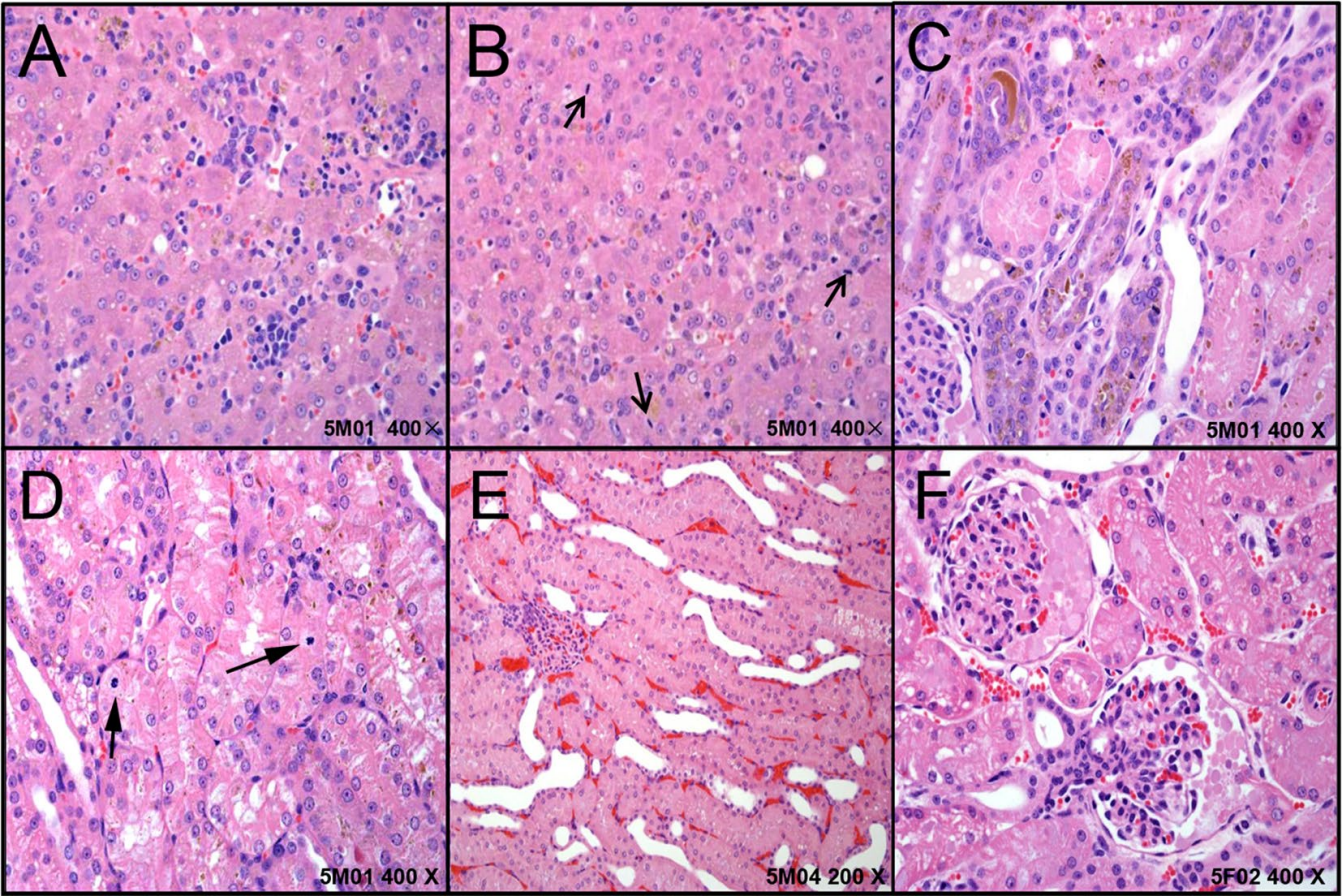
The representative histopathological changes of articulo mortis animals resulted from test articles. Hepatocyte degeneration/necrosis, and cholestasis **(A)**, inflammatory cell infiltration and nuclear mitosis **(B)**. In kidney, pigmentation and basophilic changes **(C)**, nuclear division **(D)** in the renal tubular epithelial cells, renal tubule dilatation **(E)**, and transparent droplets in the bowman capsule **(F)**.

In the digestive system, the liver in five dogs showed mild-marked hepatocyte degeneration/necrosis, minimal-slight cholestasis in four dogs (4/5) (**Figure 2A**), and minimal inflammatory cell infiltration and nuclear mitosis in three dogs (3/5) (**Figure 2B**). Mucous epithelial hyperplasia was observed in one dog’s gall bladder. In addition, three dogs’ pancreas presented minimal acinus hypertrophy. Lesions in the urinary system occurred mainly in the kidneys (slight-moderate nephrosis), such as pigmentation and basophilic changes (**Figure 2C**), nuclear division (**Figure 2D**) in the renal tubular epithelial cells, renal tubule dilatation (**Figure 2E**), and transparent droplets in the bowman capsule (**Figure 2F**). In addition, pigments were observed in one dog’s bladder.

The respiratory system injuries included moderate pneumonedema (1/5), and macrophages accumulated in pulmonary vessels (1/5). Moderate hemorrhage (1/5) and slight myocardial/interstitial vacuolation (1/5) were observed in the heart, and mild hemorrhage of the aorta occurred in one dog (1/5). Three female dogs’ uterus (3/3) and two dogs’ testis, epididymis, and prostate were immatured.

All these results demonstrated that 60 mg/kg/day arctigenin administration induced one dog death, and that the four cases of articulo mortis may have resulted from marked liver and renal injuries. In addition, one female dog (5F02) showed heart and aorta hemorrhage, and one (5F03) showed pneumonedema and macrophages accumulated, which may have resulted in articulo mortis.

### General Observation

During the drug exposure and recovery periods, animals in the control group were in good condition, with normal autonomic activities, clean coats, and no obvious toxicities. In the vehicle (PEG) group, growling, struggling, and occasional vomiting were observed during the drug exposure period. At the middle and end of the drug exposure period, animals’ skin presented with partial induration, damage, and escharosis at the injection site. All these symptoms were alleviated after the recovery period. In addition to these symptoms, 20- and 60-mg/kg/day administration in dogs resulted in sialorrhea; squatting; mental sluggishness; low food consumption; and trauma, ulceration, and escharosis the injection site. After the recovery period, all but ulcerations and escharosis still remained in the 20- and 60-mg/kg/day arctigenin treated groups, and the other dogs’ injuries resolved with no significant toxicities.

### Body Weight, Food Consumption, and Rectal Temperature

Compared with the control group, the group that received 60 mg/kg/day arctigenin had significantly decreased body weights (7.96 ± 1.02 kg vs. 9.15 ± 0.53 kg) in the third week of the drug exposure period, and the difference was eliminated after the recovery period (**Figure S2A**). In addition, the body weights among the PEG group, the arctigenin 6 mg/kg group, and the arctigenin 20 mg/kg group were not significantly different during the drug exposure and recovery periods (**Table 3**).

**Table 3:** Body weight of beagle dogs after subcutaneous injection (s.i) ARC.

Detective time	Control		PEG-treated		ARC (6 mg/kg)		ARC (20 mg/kg)		ARC (60 mg/kg)	
	n	Mean ± S.D.	n	Mean ± S.D.	n	Mean ± S.D.	n	Mean ± S.D.	n^*^	Mean ± S.D.
Before FDE	8	8.41 ± 0.41	8	8.41 ± 0.37	8	8.39 ± 0.33	8	8.48 ± 0.34	8	8.46 ± 0.25
2^nd^ day of DEP	8	8.68 ± 0.41	8	8.71 ± 0.31	8	8.68 ± 0.34	8	8.84 ± 0.34	8	8.56 ± 0.25
14^th^ day of DEP	8	8.80 ± 0.43	8	8.89 ± 0.32	8	8.85 ± 0.40	8	8.93 ± 0.52	7	8.39 ± 0.72
21^st^ day of DEP	8	9.15 ± 0.53	8	9.23 ± 0.38	8	9.14 ± 0.37	8	9.13 ± 0.51	6	7.96 ± 1.02**^#^**
28^th^ day of DEP	8	9.44 ± 0.78	8	9.74 ± 0.44	8	9.58 ± 0.50	8	9.34 ± 0.69	3	9.17 ± 1.53
7^th^ day of RP	4	9.89 ± 0.41	4	9.66 ± 0.53	4	9.53 ± 0.30	4	9.10 ± 1.29	1	8.35
14^th^ day of RP	4	10.23 ± 0.43	4	10.04 ± 0.61	4	9.88 ± 0.43	4	9.53 ± 1.08	1	8.85

FDE, first drug exposure. DEP, drug exposure period. RP, recovery period. n = 8 per treatment group, results were presented as mean ± S.D.

*After ARC (60 mg/kg) treatment, 5 animal die offs. ***^#^***Compared with control, P < 0.05.

The amount of food consumption was not different among the groups throughout the whole experiment period (*p* > 0.05). The arctigenin 60 mg/kg/day group’s food consumption showed a downward trend, but returned to normal after the recovery period (**Figure S2B**, **Table 4**).

**Table 4:** The food consumption (g/cage*24 h) of arctigenin (6, 20, and 60 mg/kg) administration by subcutaneous injection in dogs during drug exposure period (n = 8, 4 female, 4 male) and recovery period (n = 4, 2 female, 2 male per treatment group; results were presented as mean ± S.D.).

Time	Ctrl		PEG		ARC-6 mg/kg		ARC-20 mg/kg		ARC-60 mg/kg	
	n	Mean ± S.D.	n	Mean ± S.D.	n	Mean ± S.D.	n	Mean ± S.D.	n^1)^	Mean ± S.D.
Day 3	8	254.7 ± 1.7	8	255.8 ± 1.9	8	255.7 ± 2.7	8	255.3 ± 1.3	8	255.8 ± 1.8
Day 10	8	254.4 ± 1.8	8	254.5 ± 1.7	8	255.3 ± 1.8	8	253.4 ± 2.2	7	191.4 ± 84.4
Day 17	8	256.4 ± 1.6	8	255.0 ± 1.6	8	255.0 ± 1.0	8	254.3 ± 1.3	7	102.3 ± 48.5
Day 24	8	253.5 ± 1.3	8	246.7 ± 20.5	8	254.0 ± 1.5	8	254.3 ± 0.8	4	185.6 ± 80.6
Day 29	4	252.0 ± 0.8	4	252.7 ± 1.0	4	252.9 ± 1.0	4	209.7 ± 85.4	1	185.9
Day 37	4	255.1 ± 1.5	4	255.8 ± 1.6	4	255.0 ± 1.9	4	256.0 ± 1.4	1	254.9

In arctigenin-60 mg/kg treated group, four animals died (5F04/5F02/5F03/5M01) after drug exposure.

Compared with the control group, the rectal temperature was decreased in the arctigenin 6 mg/kg/day treated group (38.2± 0.1°Cvs. 38.5 ± 0.2°C. **Figure S2C**, **Table 5**). However, this fluctuation was in the normal range and not dose-dependent.

**Table 5:** The rectal temperature of arctigenin (6, 20, and 60 mg/kg) administration by subcutaneous injection in dogs during drug exposure period (n = 8, 4 female, 4 male) and recovery period (n = 4, 2 female, 2 male per treatment group; results were presented as mean ± S.D.).

Time	Ctrl		PEG		ARC-6mg/kg		ARC-20mg/kg		ARC-60mg/kg	
	n	Mean ± S.D.	n	Mean ± S.D.	n	Mean ± S.D.	n	Mean ± S.D.	n^1)^	Mean ± S.D.
2^nd^ of AP	8	38.7 ± 0.2	8	38.6 ± 0.3	8	38.5 ± 0.3	8	38.6 ± 0.3	8	38.7 ± 0.4
Before FDE	8	38.9 ± 0.2	8	38.7 ± 0.1	8	38.9 ± 0.2	8	38.8 ± 0.4	8	38.8 ± 0.1
1 h after FDE	8	38.7 ± 0.2	8	38.6 ± 0.2	8	38.8 ± 0.2	8	38.9 ± 0.3	8	38.9 ± 0.1
4 h after FDE	8	38.5 ± 0.2	8	38.5 ± 0.2	8	38.5 ± 0.2	8	38.4 ± 0.2	8	38.5 ± 0.1
24 h after FDE	8	38.7 ± 0.2	8	38.7 ± 0.3	8	38.8 ± 0.3	8	38.8 ± 0.3	8	38.7 ± 0.2
Day 14^th^ of DEP	8	38.6 ± 0.2	8	38.7 ± 0.3	8	38.8 ± 0.2	8	38.6 ± 0.2	7	38.6 ± 0.3
Day 27^th^ of DEP	8	38.5 ± 0.2	8	38.4 ± 0.3	8	38.2 ± 0.1 *	8	38.3 ± 0.2	3	38.7 ± 0.4
End of RP	4	38.9 ± 0.3	4	38.6 ± 0.2	4	38.7 ± 0.2	4	38.8 ± 0.3	1	38.6

AP, adaptive period. FDE, first drug exposure. DEP, drug exposure period. RP, recovery period.

^1)^ In arctigenin-60 mg/kg treated group, four animals died (5F04/5F02/5F03/5M01) after drug exposure.

*Compared with Ctrl, P ≤ 0.05.

In all five groups, the only death occurred in the arctigenin 60 mg/kg/day treated group (five dogs died, including one death and four articulo mortis). The survival percentage is shown in **Figure S2D**.

### Electrocardiogram

Compared with the control group, the dogs in the PEG group, arctigenin 6 mg/kg, and arctigenin 60 mg/kg group showed Q-T intermittent and shortened intervals on II lead electrocardiogram before day 14. In addition, P-wave and QRS-wave time shortened in the arctigenin 60 mg/kg treated group. However, 1 h after drug administration on day 14, all arctigenin-treated groups showed P-wave shortening. Further, 1 h after drug administration on day 20, the Q-T intermittent and correction Q-T interphase were prolonged in the arctigenin 60 mg/kg group, and P-wave altered in the arctigenin 20 mg/kg group. On day 27 (before drug exposure), the heart rates were elevated in the arctigenin 60 mg/kg group compared with the control group (**Table 6**).

**Table 6:** Electrocardiogram parameters of beagle dogs after subcutaneous injection (s.i) ARC.

Parameter time	Ctrl		PEG		ARC-6mg/kg		ARC-20mg/kg		ARC-60mg/kg	
	n	Mean ± S.D.	n	Mean ± S.D.	n	Mean ± S.D.	n	Mean ± S.D.	n^1)^	Mean ± S.D.
**HR (beats/min)**										
1^st^ in AP	8	148.68 ± 19.08	8	152.33 ± 16.46	8	143.34 ± 22.62	8	161.34 ± 22.29	8	171.23 ± 29.17
2^nd^ in AP	8	141.84 ± 21.92	8	143.16 ± 19.45	8	151.80 ± 19.08	8	164.16 ± 14.23	8	164.04 ± 14.67
Before FDE	8	123.00 ± 32.81	8	127.99 ± 16.16	8	132.29 ± 17.77	8	124.69 ± 10.70	8	132.47 ± 21.74
1 h after FDE	8	100.62 ± 24.54	8	114.34 ± 20.49	8	122.88 ± 30.76	8	138.64 ± 25.94*	8	135.00 ± 19.91*
4 h after FDE	8	108.58 ± 20.18	8	115.41 ± 20.27	8	111.24 ± 26.87	8	119.31 ± 25.14	8	124.67 ± 18.91
24 h after FDE	8	118.52 ± 21.15	8	129.04 ± 15.80	8	131.64 ± 28.11	8	138.52 ± 20.51	8	152.82 ± 25.48
Before day 14^th^ of DE	8	118.75 ± 27.07	8	127.85 ± 25.89	8	138.58 ± 25.70	8	127.49 ± 13.03	7	147.26 ± 26.70
1 h after day 14^th^ of DE	8	120.96 ± 32.04	8	123.54 ± 21.28	8	132.75 ± 21.10	8	138.43 ± 29.07	7	124.30 ± 34.22
Before day 20^th^ of DE	8	138.05 ± 18.20	8	157.56 ± 21.80	8	153.91 ± 18.83	8	144.60 ± 19.75	7	164.50 ± 22.73
1 h after day 20^th^ of DE	8	130.77 ± 39.14	8	129.71 ± 16.88	8	139.46 ± 21.94	8	145.20 ± 27.08	7	109.52 ± 35.55
2 h after day 20^th^ of DE	8	122.39 ± 18.32	8	140.66 ± 20.96	8	139.99 ± 18.90	8	129.83 ± 23.48	7	109.70 ± 45.54
Before day 27^th^ of DE	8	118.87 ± 17.06	8	133.50 ± 17.79	8	134.70 ± 27.39	8	148.55 ± 18.31*	3	166.37 ± 13.09*
1 h after day 27^th^ of DE	8	117.78 ± 24.19	8	120.13 ± 8.80	8	126.41 ± 13.15	8	132.58 ± 19.33	3	145.25 ± 41.33
End of RP	4	124.81 ± 15.42	4	148.13 ± 15.43	4	121.40 ± 14.69	4	149.67 ± 18.38	1	133.41
**R-R interval (ms)**										
1^st^ in AP	8	409.22 ± 50.66	8	397.88 ± 42.64	8	427.83 ± 67.18	8	378.38 ± 54.13	8	359.76 ± 63.11
2^nd^ in AP	8	431.30 ± 62.12	8	426.66 ± 64.13	8	401.04 ± 53.33	8	368.01 ± 33.35	8	368.27 ± 32.18
Before FDE	8	523.50 ± 158.30	8	476.72 ± 68.25	8	461.94 ± 63.77	8	485.38 ± 45.53	8	464.84 ± 78.49
1 h after FDE	8	630.88 ± 160.63	8	538.47 ± 87.83	8	512.25 ± 110.69	8	446.06 ± 83.96*	8	452.34 ± 65.81*
4 h after FDE	8	568.25 ± 107.00	8	535.41 ± 99.18	8	566.19 ± 124.99	8	523.06 ± 111.10	8	492.14 ± 86.67
24 h after FDE	8	520.47 ± 87.06	8	471.78 ± 65.36	8	471.94 ± 86.56	8	441.63 ± 66.06	8	401.52 ± 61.77*
Before day 14^th^ of DE	8	531.84 ± 137.23	8	487.13 ± 101.87	8	445.62 ± 78.91	8	474.89 ± 47.80	7	418.36 ± 71.43
1 h after day 14^th^ of DE	8	525.38 ± 129.86	8	498.79 ± 87.52	8	462.32 ± 74.90	8	449.11 ± 86.51	7	527.25 ± 189.34
Before day 20^th^ of DE	8	442.38 ± 67.61	8	387.56 ± 56.22	8	395.31 ± 51.48	8	421.25 ± 53.21	7	370.25 ± 46.68
1 h after day 20^th^ of DE	8	492.94 ± 134.24	8	470.10 ± 67.22	8	439.56 ± 68.77	8	428.78 ± 95.46	7	599.32 ± 191.93
2 h after day 20^th^ of DE	8	501.13 ± 80.49	8	435.28 ± 71.02	8	435.44 ± 59.39	8	476.78 ± 96.23	7	673.29 ± 401.60
Before day 27^th^ of DE	8	514.31 ± 76.51	8	457.03 ± 64.06	8	461.44 ± 90.89	8	408.94 ± 47.14*	3	362.08 ± 27.30*
1 h after day 27^th^ of DE	8	526.88 ± 98.10	8	502.06 ± 40.97	8	479.06 ± 48.58	8	461.38 ± 69.71	3	441.42 ± 149.49
End of RP	4	486.88 ± 66.37	4	408.25 ± 41.23	4	500.13 ± 57.58	4	405.50 ± 50.27	1	449.75
**P-wave (ms)**										
1^st^ in AP	8	48 ± 5	8	47 ± 5	8	46 ± 6	8	45 ± 5	8	44 ± 5
2^nd^ in AP	8	46 ± 8	8	45 ± 5	8	51 ± 6	8	46 ± 5	8	43 ± 4
Before FDE	8	42 ± 3	8	42 ± 3	8	43 ± 6	8	41 ± 5	8	43 ± 4
1 h after FDE	8	43 ± 4	8	41 ± 2	8	42 ± 2	8	40 ± 4	8	41 ± 3
4 h after FDE	8	41 ± 3	8	40 ± 3	8	40 ± 2	8	41 ± 4	8	39 ± 3
24 h after FDE	8	41 ± 3	8	42 ± 4	8	41 ± 4	8	40 ± 3	8	41 ± 4
Before day 14^th^ of DE	8	61 ± 12	8	68 ± 10	8	59 ± 17	8	49 ± 5	7	46 ± 5*
1 h after day 14^th^ of DE	8	61 ± 7	8	65 ± 5	8	50 ± 6 *	8	51 ± 8*	7	49 ± 5*
Before day 20^th^ of DE	8	49 ± 5	8	47 ± 4	8	50 ± 6	8	46 ± 4	7	46 ± 5
1 h after day 20^th^ of DE	8	38 ± 3	8	40 ± 3	8	38 ± 2	8	39 ± 4	7	37 ± 6
2 h after day 20^th^ of DE	8	36 ± 4	8	38 ± 4	8	40 ± 3 *	8	42 ± 2*	7	43 ± 9
Before day 27^th^ of DE	8	43 ± 5	8	42 ± 5	8	48 ± 7	8	43 ± 8	3	46 ± 3
1 h after day 27^th^ of DE	8	48 ± 6	8	49 ± 7	8	52 ± 5	8	47 ± 6	3	45 ± 4
End of RP	4	43 ± 5	4	43 ± 4	4	44 ± 3	4	43 ± 2	1	43
**P-R interval (ms)**										
1^st^ in AP	8	93 ± 10	8	87 ± 13	8	87 ± 7	8	93 ± 11	8	78 ± 8*
2^nd^ in AP	8	86 ± 11	8	88 ± 10	8	92 ± 11	8	90 ± 10	8	82 ± 8
Before FDE	8	86 ± 6	8	85 ± 11	8	85 ± 10	8	91 ± 13	8	77 ± 8
1 h after FDE	8	90 ± 8	8	90 ± 9	8	87 ± 8	8	86 ± 12	8	77 ± 12
4 h after FDE	8	85 ± 5	8	88 ± 9	8	86 ± 5	8	88 ± 9	8	76 ± 9
24 h after FDE	8	87 ± 8	8	86 ± 7	8	83 ± 7	8	93 ± 8	8	77 ± 13
Before day 14^th^ of DE	8	92 ± 13	8	102 ± 13	8	96 ± 11	8	99 ± 7	7	92 ± 10
1 h after day 14^th^ of DE	8	94 ± 11	8	101 ± 5	8	91 ± 9	8	97 ± 14	7	92 ± 11
Before day 20^th^ of DE	8	91 ± 13	8	90 ± 8	8	91 ± 4	8	99 ± 6	7	93 ± 14
1 h after day 20^th^ of DE	8	87 ± 13	8	89 ± 7	8	83 ± 6	8	92 ± 7	7	80 ± 25
2 h after day 20^th^ of DE	8	85 ± 7	8	86 ± 8	8	85 ± 6	8	93 ± 7	7	95 ± 14
Before day 27^th^ of DE	8	90 ± 10	8	86 ± 7	8	92 ± 5	8	100 ± 9	3	96 ± 13
1 h after day 27^th^ of DE	8	91 ± 8	8	94 ± 7	8	96 ± 7	8	103 ± 8	3	101 ± 25
End of RP	4	91 ± 6	4	84 ± 11	4	89 ± 5	4	95 ± 14	1	77
**QRS-wave (ms)**										
1^st^ in AP	8	53 ± 5	8	55 ± 4	8	57 ± 5	8	56 ± 4	8	53 ± 9
2^nd^ in AP	8	57 ± 5	8	55 ± 5	8	57 ± 5	8	55 ± 7	8	53 ± 6
Before FDE	8	48 ± 2	8	49 ± 3	8	50 ± 5	8	50 ± 3	8	53 ± 4
1 h after FDE	8	49 ± 5	8	49 ± 3	8	48 ± 2	8	50 ± 4	8	52 ± 3
4 h after FDE	8	50 ± 2	8	48 ± 3	8	48 ± 3	8	50 ± 2	8	51 ± 3
24 h after FDE	8	52 ± 2	8	51 ± 3	8	43 ± 9	8	49 ± 3	8	52 ± 3
Before day 14^th^ of DE	8	59 ± 7	8	53 ± 7	8	56 ± 2	8	49 ± 9	7	47 ± 9*
1 h after day 14^th^ of DE	8	56 ± 8	8	51 ± 3	8	49 ± 3	8	51 ± 3	7	54 ± 4
Before day 20^th^ of DE	8	52 ± 4	8	51 ± 3	8	50 ± 4	8	52 ± 3	7	51 ± 5
1 h after day 20^th^ of DE	8	44 ± 9	8	52 ± 7	8	44 ± 10	8	52 ± 17	7	66 ± 14*
2 h after day 20^th^ of DE	8	46 ± 13	8	41 ± 6	8	44 ± 5	8	52 ± 3	7	68 ± 23*
Before day 27^th^ of DE	8	50 ± 4	8	50 ± 3	8	50 ± 2	8	52 ± 6	3	52 ± 5
1 h after day 27^th^ of DE	8	52 ± 4	8	51 ± 4	8	51 ± 2	8	54 ± 6	3	49 ± 3
End of RP	4	51 ± 1	4	51 ± 4	4	49 ± 2	4	49 ± 3	1	56
**Q-T interval (ms)**										
1^st^ in AP	8	202 ± 35	8	185 ± 17	8	189 ± 47	8	177 ± 23	8	180 ± 31
2^nd^ in AP	8	191 ± 21	8	181 ± 19	8	169 ± 11 *	8	172 ± 17	8	164 ± 7*
Before FDE	8	187 ± 13	8	183 ± 5	8	183 ± 12	8	189 ± 8	8	185 ± 12
1 h after FDE	8	199 ± 11	8	190 ± 5	8	187 ± 14	8	186 ± 12	8	178 ± 9*
4 h after FDE	8	190 ± 9	8	191 ± 12	8	185 ± 10	8	188 ± 8	8	180 ± 8
24 h after FDE	8	184 ± 7	8	181 ± 11	8	179 ± 9	8	180 ± 7	8	173 ± 11
Before day 14^th^ of DE	8	204 ± 12	8	190 ± 12	8	183 ± 8 *	8	190 ± 13	7	179 ± 10*
1 h after day 14^th^ of DE	8	208 ± 22	8	208 ± 10	8	188 ± 13	8	185 ± 17*	7	202 ± 24
Before day 20^th^ of DE	8	188 ± 12	8	183 ± 10	8	178 ± 13	8	184 ± 10	7	187 ± 7
1 h after day 20^th^ of DE	8	182 ± 13	8	185 ± 8	8	179 ± 13	8	183 ± 10	7	222 ± 27*
2 h after day 20^th^ of DE	8	185 ± 15	8	178 ± 11	8	177 ± 8	8	185 ± 10	7	237 ± 53*
Before day 27^th^ of DE	8	190 ± 9	8	184 ± 6	8	181 ± 12	8	184 ± 8	3	170 ± 7*
1 h after day 27^th^ of DE	8	191 ± 8	8	196 ± 10	8	196 ± 8	8	196 ± 14	3	189 ± 38
End of RP	4	195 ± 12	4	192 ± 10	4	185 ± 7	4	184 ± 4	1	196
**Corrected Q-T inter. (ms)**										
1^st^ in AP	8	272 ± 41	8	252 ± 21	8	251 ± 53	8	246 ± 35	8	254 ± 35
2^nd^ in AP	8	254 ± 27	8	240 ± 17	8	229 ± 15	8	241 ± 24	8	229 ± 14
Before FDE	8	234 ± 11	8	235 ± 8	8	237 ± 7	8	240 ± 8	8	240 ± 13
1 h after FDE	8	234 ± 13	8	234 ± 14	8	235 ± 9	8	244 ± 6	8	233 ± 10
4 h after FDE	8	231 ± 10	8	235 ± 7	8	225 ± 11	8	235 ± 9	8	228 ± 7
24 h after FDE	8	230 ± 10	8	233 ± 14	8	231 ± 16	8	237 ± 7	8	235 ± 8
Before day 14^th^ of DE	8	254 ± 17	8	242 ± 10	8	241 ± 12	8	244 ± 19	7	241 ± 14
1 h after day 14^th^ of DE	8	260 ± 27	8	263 ± 13	8	244 ± 17	8	242 ± 16	7	252 ± 10
Before day 20^th^ of DE	8	247 ± 13	8	252 ± 12	8	242 ± 18	8	246 ± 10	7	261 ± 9
1 h after day 20^th^ of DE	8	232 ± 8	8	239 ± 9	8	236 ± 10	8	244 ± 11	7	265 ± 12*
2 h after day 20^th^ of DE	8	233 ± 15	8	235 ± 14	8	234 ± 9	8	238 ± 9	7	276 ± 26*
Before day 27^th^ of DE	8	238 ± 6	8	239 ± 10	8	235 ± 11	8	248 ± 11	3	239 ± 14
1 h after day 27^th^ of DE	8	238 ± 10	8	246 ± 12	8	250 ± 14	8	254 ± 13	3	249 ± 23
End of RP	4	248 ± 5	4	258 ± 12	4	233 ± 6 *	4	249 ± 6	1	256

AP, adaptive period. FDE, first drug exposure. RP, recovery period. n = 8 per treatment group, results were presented as mean ± S.D.

^1)^ In arctigenin-60 mg/kg treated group, four animals died (5F04/5F02/5F03/5M01) after drug exposure. *Compared with Ctrl, P ≤ 0.05.

The P-wave alterations were within normal ranges (within 25%), and no significant differences were found before and after drug administration. In addition, all parameters including electrocardiographic wave, HR, RR, P, PR, QRS, QT, and correction Q-T had no differences among the 1^st^, 14^th^, 20^th^, and 27^th^ day of drug exposure (*p* > 0.05).

### Hematology Examination

To investigate the potential influence of sub-chronic arctigenin administration on hematology, the parameters including RBC, HGB, HCT, MCV, MCH, MCHC, RET%, WBC, NEU, LYM, MONO, EOS, BASO, PLT, PT, and APTT were detected on days 3, 14, 27, and in the recovery period. The details of the results are shown in **Table 7**. In the early stage of the experiment (day 3), WBC, NEU, NEU%, MONO, and LYM% were elevated, and EOS%, HGB, and HCT% were reduced in the arctigenin 60 mg/kg group, compared with the control group. In the middle stage (day 14), WBC was elevated in the PEG group, WBC and NEU were elevated in the arctigenin 6 mg/kg group, and WBC, MOMO, and PLT were elevated in the arctigenin 20 mg/kg group. In addition, WBC, NEU, NEU%, MOMO, LYM%, and PLT were elevated, and PT was prolonged, in the arctigenin 60 mg/kg group. In the later stage (day 27), WBC was elevated in the PEG and arctigenin 6 mg/kg groups, and WBC, MOMO, and PLT were elevated in the arctigenin 20 mg/kg group. In addition, WBC, LYM, MOMO, RET% were elevated, HCT% and MCHC were decreased, and PT was prolonged in the arctigenin 60 mg/kg group. After a 14-day recovery period, only elevated PLT was observed in the arctigenin 20 mg/kg group.

**Table 7:** The hematological parameters of arctigenin (6, 20, and 60 mg/kg) administration by subcutaneous injection in dogs during drug exposure period (n = 8, 4 female, 4 male) and recovery period (n = 4, 2 female, 2 male per treatment group, results were presented as mean ± S.D.).

Paremeters Detect Time	Control		PEG-group		ARC-6 mg/kg group		ARC-20 mg/kg group		ARC-60 mg/kg group	
	n	Mean ± SD	n	Mean ± SD	n	Mean ± SD	n	Mean ± SD	n^1)^	Mean ± SD
**WBC (10** **^9^** **/L)**										
The 1^st^ detect in AP	8	12.15 ± 2.31	8	13.97 ± 3.75	8	13.61 ± 3.40	8	11.25 ± 1.40	8	11.61 ± 2.57
The 2^nd^ detect in AP	8	12.12 ± 1.61	8	11.28 ± 1.67	8	13.02 ± 2.08	8	11.18 ± 1.23	8	12.13 ± 3.26
Day-3 (Before DD)	8	10.56 ± 2.16	8	12.07 ± 1.63	8	13.33 ± 2.58	8	11.93 ± 1.94	8	19.10 ± 3.17*
Day-14 (Before DD)	8	9.10 ± 0.91	8	11.57 ± 2.67	8	11.71 ± 2.19*	8	10.75 ± 1.53*	7	22.99 ± 8.43*
Day-27 (Before DD)	8	10.82 ± 1.39	8	14.00 ± 2.91*	8	12.44 ± 0.71*	8	14.83 ± 3.62*	3	23.52 ± 7.74*
End of RP	4	12.99 ± 2.73	4	14.80 ± 4.23	4	12.81 ± 1.87	4	14.73 ± 4.12	1	23.35
**NEU (10** **^9^** **/L)**										
The 1^st^ detect in AP	8	7.61 ± 1.84	8	8.76 ± 2.49	8	8.14 ± 2.71	8	6.41 ± 1.03	8	6.74 ± 2.06
The 2^nd^ detect in AP	8	7.66 ± 1.05	8	6.69 ± 1.48	8	7.45 ± 1.61	8	6.40 ± 0.79	8	7.27 ± 2.64
Day-3 (Before DD)	8	6.55 ± 1.70	8	7.44 ± 1.33	8	8.41 ± 2.23	8	7.66 ± 2.07	8	14.42 ± 2.87*
Day-14 (Before DD)	8	5.44 ± 0.76	8	7.42 ± 2.18	8	7.31 ± 2.15*	8	6.56 ± 1.43	7	17.50 ± 7.70*
Day-27 (Before DD)	8	7.06 ± 1.39	8	9.27 ± 2.47	8	7.59 ± 0.73	8	9.74 ± 3.31	3	14.72 ± 7.42
End of RP	4	8.25 ± 2.13	4	10.14 ± 3.17	4	7.37 ± 1.42	4	10.20 ± 3.54	1	17.02
**LYM (10** **^9^** **/L)**										
The 1^st^ detect in AP	8	3.40 ± 0.55	8	3.88 ± 1.18	8	4.02 ± 0.58	8	3.64 ± 0.80	8	3.64 ± 0.78
The 2^nd^ detect in AP	8	3.39 ± 0.54	8	3.50 ± 0.52	8	4.25 ± 0.74	8	3.65 ± 0.74	8	3.68 ± 0.90
Day-3 (Before DD)	8	2.99 ± 0.50	8	3.40 ± 0.44	8	3.53 ± 0.28	8	3.10 ± 0.42	8	3.15 ± 0.42
Day-14 (Before DD)	8	2.84 ± 0.62	8	3.12 ± 0.52	8	3.29 ± 0.41	8	3.03 ± 0.68	7	3.70 ± 0.80
Day-27 (Before DD)	8	2.74 ± 0.58	8	3.38 ± 0.62	8	3.53 ± 0.53	8	3.57 ± 0.97	3	7.18 ± 1.01*
End of RP	4	3.65 ± 0.48	4	3.24 ± 0.63	4	3.85 ± 0.21	4	3.27 ± 0.36	1	3.58
**MONO (10** **^9^** **/L)**										
The 1^st^ detect in AP	8	0.74 ± 0.21	8	0.83 ± 0.29	8	0.92 ± 0.33	8	0.83 ± 0.19	8	0.75 ± 0.19
The 2^nd^ detect in AP	8	0.69 ± 0.20	8	0.60 ± 0.15	8	0.73 ± 0.17	8	0.68 ± 0.25	8	0.62 ± 0.16
Day-3 (Before DD)	8	0.68 ± 0.19	8	0.69 ± 0.14	8	0.88 ± 0.31	8	0.83 ± 0.16	8	1.15 ± 0.31*
Day-14 (Before DD)	8	0.47 ± 0.11	8	0.57 ± 0.15	8	0.64 ± 0.25	8	0.73 ± 0.23 *	7	1.30 ± 0.36*
Day-27 (Before DD)	8	0.64 ± 0.16	8	0.79 ± 0.22	8	0.73 ± 0.20	8	1.06 ± 0.38 *	3	1.20 ± 0.24*
End of RP	4	0.59 ± 0.24	4	0.66 ± 0.40	4	0.70 ± 0.32	4	0.92 ± 0.21	1	2.09
**EOS (10** **^9^** **/L)**										
The 1^st^ detect in AP	8	0.27 ± 0.12	8	0.34 ± 0.16	8	0.36 ± 0.14	8	0.21 ± 0.10	8	0.34 ± 0.15
The 2^nd^ detect in AP	8	0.24 ± 0.11	8	0.36 ± 0.24	8	0.40 ± 0.27	8	0.28 ± 0.12	8	0.42 ± 0.21
Day-3 (Before DD)	8	0.21 ± 0.11	8	0.42 ± 0.31	8	0.34 ± 0.28	8	0.21 ± 0.16	8	0.16 ± 0.14
Day-14 (Before DD)	8	0.20 ± 0.12	8	0.28 ± 0.18	8	0.29 ± 0.21	8	0.23 ± 0.13	7	0.27 ± 0.08
Day-27 (Before DD)	8	0.26 ± 0.09	8	0.41 ± 0.27	8	0.42 ± 0.30	8	0.29 ± 0.16	3	0.25 ± 0.19
End of RP	4	0.38 ± 0.10	4	0.62 ± 0.41	4	0.77 ± 0.40	4	0.26 ± 0.06	1	0.55
**BASO (10** **^9^** **/L)**										
The 1^st^ detect in AP	8	0.06 ± 0.01	8	0.07 ± 0.03	8	0.06 ± 0.01	8	0.07 ± 0.02	8	0.06 ± 0.02
The 2^nd^ detect in AP	8	0.05 ± 0.01	8	0.06 ± 0.02	8	0.08 ± 0.02 *	8	0.07 ± 0.01	8	0.05 ± 0.02
Day-3 (Before DD)	8	0.05 ± 0.01	8	0.06 ± 0.02	8	0.06 ± 0.02	8	0.05 ± 0.01	8	0.05 ± 0.01
Day-14 (Before DD)	8	0.07 ± 0.03	8	0.07 ± 0.03	8	0.08 ± 0.02	8	0.08 ± 0.02	7	0.09 ± 0.05
Day-27 (Before DD)	8	0.06 ± 0.02	8	0.08 ± 0.02	8	0.08 ± 0.04	8	0.10 ± 0.04	3	0.09 ± 0.05
End of RP	4	0.06 ± 0.03	4	0.07 ± 0.04	4	0.06 ± 0.02	4	0.05 ± 0.01	1	0.04
**NEU % (%)**										
The 1^st^ detect in AP	8	62.1 ± 5.1	8	62.5 ± 3.1	8	59.0 ± 5.0	8	57.1 ± 6.3	8	57.2 ± 6.7
The 2^nd^ detect in AP	8	63.3 ± 3.1	8	58.8 ± 5.2	8	57.0 ± 5.4	8	57.4 ± 4.6	8	58.9 ± 7.4
Day-3 (Before DD)	8	61.6 ± 5.4	8	61.4 ± 4.5	8	62.4 ± 5.5	8	63.4 ± 8.0	8	75.2 ± 3.4*
Day-14 (Before DD)	8	59.7 ± 6.0	8	63.4 ± 4.7	8	61.4 ± 8.1	8	60.9 ± 6.9	7	74.5 ± 6.5*
Day-27 (Before DD)	8	64.8 ± 6.1	8	65.6 ± 5.5	8	61.0 ± 4.5	8	64.8 ± 9.1	3	59.5 ± 15.1
End of RP	4	63.1 ± 3.5	4	68.3 ± 3.2	4	57.3 ± 2.6	4	68.2 ± 5.1	1	72.9
**LYM % (%)**										
The 1^st^ detect in AP	8	28.4 ± 4.3	8	27.8 ± 3.8	8	30.4 ± 4.9	8	32.3 ± 5.0	8	31.9 ± 5.1
The 2^nd^ detect in AP	8	28.0 ± 2.5	8	31.3 ± 4.3	8	33.0 ± 5.4	8	32.6 ± 5.1	8	31.3 ± 6.9
Day-3 (Before DD)	8	28.7 ± 4.5	8	28.2 ± 2.7	8	27.2 ± 4.6	8	26.5 ± 5.7	8	16.8 ± 2.5*
Day-14 (Before DD)	8	31.1 ± 5.7	8	27.7 ± 4.5	8	28.9 ± 6.8	8	28.4 ± 6.3	7	17.3 ± 5.2*
Day-27 (Before DD)	8	25.8 ± 6.3	8	24.7 ± 5.2	8	28.4 ± 4.0	8	24.9 ± 7.0	3	33.4 ± 14.5
End of RP	4	28.5 ± 3.2	4	22.4 ± 2.9	4	30.5 ± 4.6	4	23.0 ± 4.2	1	15.3
**MONO % (%)**										
The 1^st^ detect in AP	8	6.1 ± 0.9	8	6.1 ± 1.7	8	6.7 ± 1.2	8	7.4 ± 1.6	8	6.8 ± 2.2
The 2^nd^ detect in AP	8	5.7 ± 1.3	8	5.4 ± 1.4	8	5.5 ± 0.7	8	6.1 ± 1.8	8	5.2 ± 0.9
Day-3 (Before DD)	8	6.4 ± 0.9	8	5.7 ± 1.1	8	6.5 ± 1.5	8	7.1 ± 1.8	8	6.1 ± 1.7
Day-14 (Before DD)	8	5.2 ± 1.4	8	5.0 ± 1.2	8	5.3 ± 1.4	8	6.7 ± 1.2	7	5.9 ± 1.4
Day-27 (Before DD)	8	6.0 ± 1.3	8	5.7 ± 1.6	8	5.9 ± 1.5	8	7.1 ± 2.0	3	5.3 ± 0.8
End of RP	4	4.5 ± 1.3	4	4.2 ± 1.3	4	5.4 ± 1.8	4	6.3 ± 0.6	1	9.0
**EOS % (%)**										
The 1^st^ detect in AP	8	2.3 ± 1.2	8	2.6 ± 1.2	8	2.7 ± 0.9	8	1.9 ± 0.7	8	3.0 ± 1.1
The 2^nd^ detect in AP	8	2.0 ± 0.9	8	3.3 ± 2.4	8	3.0 ± 1.4	8	2.5 ± 0.9	8	3.3 ± 1.3
Day-3 (Before DD)	8	2.1 ± 1.2	8	3.5 ± 2.7	8	2.6 ± 2.1	8	1.8 ± 1.4	8	0.8 ± 0.8 *
Day-14 (Before DD)	8	2.2 ± 1.2	8	2.5 ± 1.4	8	2.7 ± 2.0	8	2.2 ± 1.3	7	1.3 ± 0.5
Day-27 (Before DD)	8	2.4 ± 0.8	8	3.0 ± 1.7	8	3.4 ± 2.3	8	2.1 ± 1.2	3	1.0 ± 0.7
End of RP	4	3.0 ± 1.0	4	4.2 ± 2.5	4	5.9 ± 2.8	4	1.8 ± 0.1	1	2.3
**BASO % (%)**										
The 1^st^ detect in AP	8	0.5 ± 0.1	8	0.5 ± 0.1	8	0.5 ± 0.2	8	0.6 ± 0.2	8	0.6 ± 0.2
The 2^nd^ detect in AP	8	0.5 ± 0.1	8	0.5 ± 0.1	8	0.6 ± 0.2	8	0.6 ± 0.1	8	0.5 ± 0.2
Day-3 (Before DD)	8	0.5 ± 0.1	8	0.5 ± 0.1	8	0.5 ± 0.2	8	0.5 ± 0.1	8	0.3 ± 0.1 *
Day-14 (Before DD)	8	0.8 ± 0.4	8	0.6 ± 0.2	8	0.7 ± 0.2	8	0.8 ± 0.2	7	0.4 ± 0.2 *
Day-27 (Before DD)	8	0.6 ± 0.2	8	0.5 ± 0.1	8	0.7 ± 0.3	8	0.7 ± 0.3	3	0.4 ± 0.2
End of RP	4	0.4 ± 0.2	4	0.4 ± 0.2	4	0.5 ± 0.1	4	0.3 ± 0.2	1	0.2
**RBC (10** **^12^** **/L)**										
The 1^st^ detect in AP	8	6.38 ± 0.57	8	6.79 ± 0.29	8	6.71 ± 0.26	8	6.57 ± 0.37	8	6.62 ± 0.45
The 2^nd^ detect in AP	8	6.61 ± 0.37	8	7.06 ± 0.49	8	6.87 ± 0.39	8	6.76 ± 0.30	8	6.50 ± 0.42
Day-3 (Before DD)	8	6.33 ± 0.53	8	6.45 ± 0.35	8	6.21 ± 0.40	8	5.99 ± 0.27	8	5.88 ± 0.65
Day-14 (Before DD)	8	6.31 ± 0.72	8	6.12 ± 0.42	8	6.13 ± 0.49	8	6.36 ± 0.19	7	6.25 ± 0.63
Day-27 (Before DD)	8	6.57 ± 0.51	8	6.67 ± 0.67	8	6.74 ± 0.51	8	6.79 ± 0.41	3	4.84 ± 1.22
End of RP	4	6.51 ± 0.15	4	6.75 ± 0.48	4	6.75 ± 0.45	4	5.96 ± 0.77	1	4.77
**HGB (g/L)**										
The 1^st^ detect in AP	8	136 ± 11	8	142 ± 6	8	140 ± 6	8	137 ± 10	8	138 ± 10
The 2^nd^ detect in AP	8	141 ± 6	8	148 ± 11	8	145 ± 8	8	142 ± 7	8	137 ± 10
Day-3 (Before DD)	8	139 ± 10	8	138 ± 8	8	135 ± 7	8	129 ± 8	8	125 ± 15 *
Day-14 (Before DD)	8	140 ± 14	8	134 ± 9	8	135 ± 9	8	140 ± 5	7	134 ± 12
Day-27 (Before DD)	8	143 ± 9	8	145 ± 14	8	148 ± 10	8	147 ± 9	3	104 ± 27
End of RP	4	143 ± 5	4	146 ± 12	4	145 ± 8	4	125 ± 19	1	103
**HCT (%)**										
The 1^st^ detect in AP	8	42.5 ± 3.3	8	44.9 ± 2.0	8	44.0 ± 1.8	8	43.2 ± 2.8	8	43.3 ± 2.9
The 2^nd^ detect in AP	8	44.3 ± 1.9	8	46.6 ± 3.3	8	45.5 ± 2.9	8	45.0 ± 2.5	8	43.0 ± 3.0
Day-3 (Before DD)	8	42.8 ± 3.0	8	43.0 ± 2.5	8	41.5 ± 2.2	8	40.0 ± 2.1	8	38.7 ± 4.0 *
Day-14 (Before DD)	8	43.5 ± 4.2	8	41.7 ± 2.9	8	41.9 ± 2.9	8	44.0 ± 1.6	7	41.9 ± 4.7
Day-27 (Before DD)	8	44.4 ± 2.5	8	44.7 ± 4.5	8	45.1 ± 2.9	8	46.1 ± 2.7	3	34.8 ± 5.5 *
End of RP	4	45.2 ± 2.1	4	46.1 ± 3.5	4	46.0 ± 2.3	4	40.2 ± 5.8	1	37.0
**MCV (fL)**										
The 1^st^ detect in AP	8	66.7 ± 1.5	8	66.1 ± 2.1	8	65.6 ± 2.0	8	65.8 ± 2.1	8	65.4 ± 1.9
The 2^nd^ detect in AP	8	67.1 ± 1.5	8	65.9 ± 1.7	8	66.3 ± 1.8	8	66.5 ± 1.6	8	66.2 ± 2.0
Day-3 (Before DD)	8	67.6 ± 1.5	8	66.8 ± 1.7	8	66.8 ± 1.7	8	66.7 ± 1.9	8	65.9 ± 1.5
Day-14 (Before DD)	8	69.1 ± 1.6	8	68.1 ± 1.6	8	68.4 ± 1.6	8	69.2 ± 1.6	7	67.0 ± 2.6
Day-27 (Before DD)	8	67.8 ± 1.8	8	66.9 ± 1.7	8	67.1 ± 1.6	8	67.9 ± 1.8	3	73.2 ± 8.6
End of RP	4	69.4 ± 1.8	4	68.4 ± 2.0	4	68.4 ± 1.9	4	67.3 ± 1.6	1	77.6
**MCH (pg)**										
The 1^st^ detect in AP	8	21.2 ± 0.5	8	20.9 ± 0.6	8	20.9 ± 0.5	8	20.9 ± 0.5	8	20.7 ± 0.4
The 2^nd^ detect in AP	8	21.3 ± 0.5	8	21.0 ± 0.5	8	21.1 ± 0.4	8	21.0 ± 0.5	8	21.1 ± 0.5
Day-3 (Before DD)	8	21.9 ± 0.5	8	21.5 ± 0.5	8	21.8 ± 0.6	8	21.5 ± 0.7	8	21.1 ± 0.4
Day-14 (Before DD)	8	22.3 ± 0.6	8	21.8 ± 0.5	8	22.0 ± 0.6	8	22.0 ± 0.5	7	21.5 ± 0.6
Day-27 (Before DD)	8	21.9 ± 0.5	8	21.7 ± 0.6	8	22.0 ± 0.5	8	21.7 ± 0.7	3	21.4 ± 0.7
End of RP	4	22.0 ± 0.4	4	21.6 ± 0.6	4	21.6 ± 0.6	4	21.0 ± 0.5	1	21.7
**MCHC (g/L)**										
The 1^st^ detect in AP	8	318 ± 4	8	315 ± 3	8	319 ± 5	8	317 ± 5	8	317 ± 6
The 2^nd^ detect in AP	8	317 ± 2	8	318 ± 4	8	319 ± 4	8	316 ± 3	8	318 ± 4
Day-3 (Before DD)	8	324 ± 2	8	322 ± 4	8	326 ± 3	8	322 ± 5	8	321 ± 7
Day-14 (Before DD)	8	322 ± 3	8	320 ± 3	8	322 ± 3	8	318 ± 3	7	321 ± 8
Day-27 (Before DD)	8	323 ± 4	8	324 ± 3	8	328 ± 3 *	8	319 ± 5	3	295 ± 33 *
End of RP	4	317 ± 3	4	316 ± 4	4	315 ± 3	4	311 ± 6	1	279
**RET % (%)**										
The 1^st^ detect in AP	8	1.72 ± 0.47	8	2.17 ± 0.94	8	1.65 ± 0.21	8	1.85 ± 0.70	8	1.95 ± 0.62
The 2^nd^ detect in AP	8	2.36 ± 0.75	8	2.30 ± 0.62	8	2.15 ± 0.44	8	2.21 ± 0.62	8	2.54 ± 0.75
Day-3 (Before DD)	8	1.06 ± 0.36	8	1.13 ± 0.52	8	1.06 ± 0.32	8	0.82 ± 0.23	8	0.82 ± 0.13
Day-14 (Before DD)	8	1.34 ± 0.46	8	1.49 ± 0.52	8	1.40 ± 0.35	8	1.29 ± 0.58	7	0.98 ± 0.62
Day-27 (Before DD)	8	1.16 ± 0.49	8	1.38 ± 0.51	8	0.98 ± 0.24	8	1.16 ± 0.69	3	7.59 ± 8.70 *
End of RP	4	1.58 ± 0.35	4	1.64 ± 0.66	4	1.54 ± 0.65	4	2.16 ± 1.77	1	10.52
**PLT (10** **^9^** **/L)**										
The 1^st^ detect in AP	8	584 ± 119	8	501 ± 114	8	472 ± 70	8	542 ± 67	8	569 ± 45
The 2^nd^ detect in AP	8	559 ± 129	8	474 ± 101	8	450 ± 81	8	527 ± 88	8	504 ± 69
Day-3 (Before DD)	8	449 ± 71	8	382 ± 76	8	374 ± 48	8	424 ± 105	8	349 ± 40
Day-14 (Before DD)	8	411 ± 55	8	376 ± 73	8	381 ± 47	8	562 ± 93 *	7	588 ± 67 *
Day-27 (Before DD)	8	411 ± 96	8	389 ± 59	8	392 ± 48	8	687 ± 86 *	3	736 ± 157 *
End of RP	4	383 ± 78	4	333 ± 80	4	350 ± 29	4	641 ± 227 *	1	1022
**PT (s)**										
The 1^st^ detect in AP	8	6.3 ± 0.5	8	6.5 ± 1.0	8	6.3 ± 0.3	8	8.6 ± 6.3	8	6.8 ± 1.0
The 2^nd^ detect in AP	8	6.8 ± 0.6	8	6.9 ± 1.0	8	6.7 ± 0.3	8	9.4 ± 7.4	8	7.2 ± 1.1
Day-3 (Before DD)	8	7.4 ± 1.0	8	7.0 ± 1.1	8	6.7 ± 0.2	8	9.2 ± 6.5	8	7.3 ± 1.1
Day-14 (Before DD)	8	6.7 ± 0.7	8	6.6 ± 1.0	8	6.3 ± 0.2	8	8.5 ± 5.1	7	15.5 ± 9.9 *
Day-27 (Before DD)	8	6.6 ± 0.5	8	6.6 ± 1.0	8	6.2 ± 0.3	8	9.9 ± 5.3	3	11.6 ± 2.3 *
End of RP	4	6.3 ± 0.4	4	6.4 ± 0.4	4	6.0 ± 0.1	4	6.4 ± 0.9	1	6.4
**APTT (s)**										
The 1^st^ detect in AP	8	8.1 ± 0.6	8	7.8 ± 0.7	8	8.3 ± 0.7	8	8.0 ± 0.3	8	8.0 ± 0.5
The 2^nd^ detect in AP	8	7.9 ± 0.7	8	7.9 ± 0.7	8	8.3 ± 0.7	8	8.1 ± 0.5	8	8.2 ± 0.6
Day-3 (Before DD)	8	7.4 ± 0.7	8	7.8 ± 1.3	8	8.1 ± 0.6	8	7.9 ± 0.6	8	8.4 ± 0.9
Day-14 (Before DD)	8	7.4 ± 0.4	8	7.4 ± 0.9	8	7.3 ± 0.5	8	7.0 ± 0.6	7	8.5 ± 3.4
Day-27 (Before DD)	8	7.8 ± 0.5	8	7.4 ± 1.0	8	7.2 ± 0.6	8	6.8 ± 1.7	3	8.7 ± 2.7
End of RP	4	7.4 ± 0.6	4	6.6 ± 0.5	4	7.0 ± 0.3	4	7.9 ± 1.0	1	8.4

AP, adaptation period; DD, drug delivery; RP, recovery period; WBC, white blood cell; NEU, neutrophil; LYM, lymphocyte; MONO, monocyte; EOS, eosinophil; BASO, basophil; RBC, red blood cell; HGB, hemoglobin; HCT %, hematocrit; MCV, mean corpuscular volume; MCH, mean corpuscular hemoglobin; MCHC, mean corpuscular hemoglobin concentration; RET %, reticulocytes; 1). Arctigenin 60 mg/kg group, four dogs (5F04, 5F02, 5F03, and 5M01) successively died, resulting in animal sample reduction. *Compared with control group, P ≤ 0.05.

### Blood Biochemical Examination

Compared with the control group at the same time point, PEG treatment induced a decrease in TBIL at day 27. However, arctigenin administration resulted in a series of changes as follows: 1) On day 3, CK increased, and TBIL and K^+^ decreased by arctigenin 6 mg/kg treatment; GLU and Cl^−^ increased, and AST, CK-MB, LDH, and CHOL decreased by arctigenin 20 mg/kg treatment; GLU and Cl^−^ increased, and AST, GGT, CK-MB, LDH, urea, crea, TP, ALB, TBIL, K^+^, Ca, and AMY-P decreased by arctigenin 60 mg/kg treatment. 2) On day 14, K^+^ decreased by arctigenin 6 mg/kg treatment; ALP, ALT, AST, and Cl^−^ increased, and ALB decreased by arctigenin 20 mg/kg treatment; ALP, ALT, and AST increased, and crea, TP, ALB, CHOL, K^+^, Ca, and P decreased by arctigenin 60 mg/kg treatment. 3) On day 27, TBIL decreased by arctigenin 6 mg/kg treatment; ALP, ALT, AST, and GGT increased; ALB and CHOL decreased by arctigenin 20 mg/kg treatment; ALP, ALT, AST, GGT, LDH, TBIL, Cl^−^, and LIPC increased, and crea, TP, ALB, A/G, CHOL, Ca, and AMY-P decreased by arctigenin 60 mg/kg treatment. 4) At the end of the recovery period, crea and ALB were reduced by arctigenin 20 mg/kg treatment.

Combined with individual parameters, the amplitude of TP, K^+^, Cl^−^, Ca, P, AMY-P, crea, and ALB variations was less than 35%, and was not time- and dose-dependent, which suggests that all these changes were within normal limits. In contrast, ALP, ALT, AST, and GGT were significantly increased and CHOL was decreased by 20 mg/kg treatment. ALP, ALT, AST, TBIL, GGT, and LIPC were significantly elevated and ALB and A/G were decreased by 60 mg/kg treatment. These changes were time- and dose-dependent, which suggests that they may have resulted from arctigenin administration. In addition, there were no differences in any of the parameters during the drug exposure and recovery periods. The data are shown in **Table 8**.

**Table 8:** The blood biochemistry parameters of arctigenin (6, 20, and 60 mg/kg) administration by subcutaneous injection in dogs during drug exposure period (n = 8, 4 female, 4 male) and recovery period (n=4, 2 female, 2 male per treatment group; results were presented as mean ± S.D.).

Paremeters Detect time	Control		PEG-group		ARC-6 mg/kg group		ARC-20 mg/kg group		ARC-60 mg/kg group	
	n	Mean ± SD	n	Mean ± SD	n	Mean ± SD	n	Mean ± SD	n^1)^	Mean ± SD
**ALP (U/L)**										
The 1^st^ detect in AP	8	139.8 ± 46.6	8	136.7 ± 26.7	8	139.2 ± 41.2	8	184.3 ± 85.0	8	122.8 ± 19.3
The 2^nd^ detect in AP	8	149.7 ± 60.8	8	126.5 ± 34.9	8	145.3 ± 45.7	8	181.8 ± 71.7	8	137.8 ± 24.1
Day-3 (Before DD)	8	143.0 ± 64.7	8	120.2 ± 23.9	8	130.5 ± 43.0	8	157.8 ± 68.2	8	141.6 ± 55.0
Day-14 (Before DD)	8	133.7 ± 68.4	8	110.8 ± 20.4	8	127.4 ± 40.9	8	208.0 ± 66.8*	7	1066.3 ± 810.0*
Day-27 (Before DD)	8	129.3 ± 63.2	8	101.6 ± 18.1	8	121.6 ± 35.8	8	542.2 ± 372.3*	3	1333.6 ± 202.1*
End of RP	4	145.7 ± 84.0	4	103.0 ± 34.7	4	109.4 ± 29.4	4	195.1 ± 107.1	1	539.2
**ALT (U/L)**										
The 1^st^ detect in AP	8	26.7 ± 8.0	8	26.7 ± 6.0	8	25.9 ± 8.1	8	27.4 ± 3.8	8	24.9 ± 3.8
The 2^nd^ detect in AP	8	23.9 ± 4.2	8	27.4 ± 5.6	8	24.3 ± 6.4	8	27.1 ± 3.4	8	25.3 ± 3.9
Day-3 (Before DD)	8	28.9 ± 4.6	8	34.0 ± 8.9	8	33.4 ± 9.0	8	34.3 ± 5.8	8	33.6 ± 5.3
Day-14 (Before DD)	8	28.2 ± 3.8	8	35.6 ± 11.7	8	32.0 ± 6.2	8	46.9 ± 15.9*	7	362.4 ± 421.6*
Day-27 (Before DD)	8	31.0 ± 4.1	8	31.7 ± 7.9	8	31.9 ± 6.5	8	223.7 ± 269.0*	3	312.5 ± 127.9*
End of RP	4	29.0 ± 6.3	4	29.8 ± 5.5	4	26.1 ± 7.3	4	36.8 ± 13.3	1	35.5
**AST (U/L)**										
The 1^st^ detect in AP	8	32.8 ± 4.9	8	31.9 ± 3.3	8	29.3 ± 4.5	8	32.7 ± 4.0	8	34.8 ± 4.4
The 2^nd^ detect in AP	8	32.9 ± 4.3	8	32.1 ± 4.1	8	30.7 ± 3.3	8	31.1 ± 4.3	8	31.7 ± 2.8
Day-3 (Before DD)	8	33.5 ± 10.8	8	30.4 ± 4.1	8	35.1 ± 5.3	8	30.0 ± 2.5	8	25.4 ± 1.9 *
Day-14 (Before DD)	8	29.8 ± 4.1	8	30.2 ± 6.0	8	28.4 ± 2.8	8	35.9 ± 9.4	7	345.8 ± 475.1 *
Day-27 (Before DD)	8	32.8 ± 4.4	8	31.9 ± 5.7	8	31.9 ± 2.3	8	139.9 ± 212.8	3	196.0 ± 97.6 *
End of RP	4	33.1 ± 4.8	4	34.0 ± 4.0	4	30.7 ± 1.9	4	29.2 ± 2.6	1	28.7
**GGT(U/L)**										
The 1^st^ detect in AP	8	4.9 ± 1.0	8	3.9 ± 0.6	8	3.8 ± 1.1	8	4.0 ± 1.4	8	3.4 ± 0.4
The 2^nd^ detect in AP	8	4.4 ± 1.0	8	3.4 ± 0.9	8	4.4 ± 0.9	8	4.1 ± 1.7	8	3.6 ± 0.5
Day-3 (Before DD)	8	5.2 ± 1.6	8	3.9 ± 0.8	8	3.8 ± 0.9	8	4.2 ± 1.6	8	3.3 ± 0.7*
Day-14 (Before DD)	8	4.0 ± 1.2	8	3.1 ± 0.3	8	3.6 ± 0.9	8	3.8 ± 1.1	7	5.4 ± 3.6
Day-27 (Before DD)	8	4.0 ± 1.1	8	3.8 ± 0.4	8	4.5 ± 1.0	8	6.7 ± 3.3*	3	6.6 ± 1.7*
End of RP	4	3.4 ± 2.4	4	3.1 ± 1.3	4	3.6 ± 1.7	4	2.9 ± 1.1	1	4.5
**CK (U/L)**										
The 1^st^ detect in AP	8	256 ± 44	8	264 ± 49	8	228 ± 36	8	278 ± 46	8	318 ± 49*
The 2^nd^ detect in AP	8	287 ± 39	8	275 ± 58	8	246 ± 27	8	253 ± 46	8	287 ± 34
Day-3 (Before DD)	8	366 ± 272	8	303 ± 73	8	882 ± 476*	8	437 ± 137	8	364 ± 84
Day-14 (Before DD)	8	215 ± 27	8	217 ± 37	8	225 ± 55	8	312 ± 195	7	755 ± 979
Day-27 (Before DD)	8	230 ± 50	8	242 ± 61	8	236 ± 98	8	216 ± 73	3	276 ± 37
End of RP	4	201 ± 33	4	199 ± 45	4	196 ± 6	4	190 ± 27	1	163
**CK-MB (U/L)**										
The 1^st^ detect in AP	8	304 ± 70	8	309 ± 46	8	293 ± 48	8	306 ± 44	8	354 ± 73
The 2^nd^ detect in AP	8	339 ± 83	8	323 ± 48	8	304 ± 38	8	294 ± 44	8	327 ± 49
Day-3 (Before DD)	8	325 ± 62	8	304 ± 38	8	290 ± 43	8	253 ± 28*	8	199 ± 26*
Day-14 (Before DD)	8	280 ± 43	8	285 ± 47	8	248 ± 35	8	241 ± 31	7	303 ± 135
Day-27 (Before DD)	8	272 ± 41	8	268 ± 47	8	227 ± 20	8	267 ± 107	3	332 ± 92
End of RP	4	269 ± 52	4	278 ± 57	4	235 ± 5	4	272 ± 59	1	246
**LDH(U/L)**										
The 1^st^ detect in AP	8	79 ± 22	8	80 ± 27	8	68 ± 12	8	81 ± 24	8	86 ± 27
The 2^nd^ detect in AP	8	94 ± 25	8	86 ± 22	8	76 ± 15	8	80 ± 33	8	88 ± 34
Day-3 (Before DD)	8	117 ± 41	8	88 ± 24	8	85 ± 22	8	68 ± 8*	8	58 ± 13*
Day-14 (Before DD)	8	71 ± 18	8	77 ± 17	8	67 ± 17	8	62 ± 10	7	130 ± 119
Day-27 (Before DD)	8	79 ± 20	8	83 ± 27	8	60 ± 8	8	87 ± 53	3	166 ± 46*
End of RP	4	66 ± 31	4	70 ± 31	4	50 ± 6	4	79 ± 44	1	49
**Urea (タµmol/L)**										
The 1^st^ detect in AP	8	3.16 ± 0.63	8	3.12 ± 0.36	8	3.18 ± 0.57	8	2.64 ± 0.69	8	3.36 ± 0.78
The 2^nd^ detect in AP	8	3.64 ± 0.63	8	3.45 ± 0.29	8	3.37 ± 0.42	8	2.94 ± 0.49	8	3.34 ± 0.58
Day-3 (Before DD)	8	3.26 ± 0.61	8	2.93 ± 0.46	8	2.80 ± 0.59	8	2.64 ± 0.63	8	2.39 ± 0.53*
Day-14 (Before DD)	8	3.31 ± 0.72	8	3.20 ± 0.46	8	3.34 ± 0.47	8	2.75 ± 0.76	7	3.00 ± 1.14
Day-27 (Before DD)	8	3.50 ± 0.63	8	3.09 ± 0.44	8	3.49 ± 0.42	8	2.86 ± 0.69	3	3.02 ± 0.74
End of RP	4	4.24 ± 0.78	4	3.39 ± 0.28	4	4.30 ± 0.83	4	3.53 ± 0.17	1	3.43
**Crea (タµmol/L)**										
The 1^st^ detect in AP	8	41.5 ± 5.4	8	42.3 ± 4.9	8	44.0 ± 2.2	8	38.4 ± 3.8	8	42.4 ± 6.6
The 2^nd^ detect in AP	8	42.4 ± 5.5	8	43.4 ± 5.9	8	45.4 ± 4.1	8	38.8 ± 4.7	8	40.6 ± 4.9
Day-3 (Before DD)	8	44.8 ± 6.0	8	42.3 ± 3.5	8	45.7 ± 3.4	8	39.7 ± 4.5	8	33.5 ± 6.5*
Day-14 (Before DD)	8	50.9 ± 6.1	8	48.6 ± 5.0	8	53.9 ± 2.8	8	44.6 ± 5.6	7	35.6 ± 7.2*
Day-27 (Before DD)	8	50.2 ± 5.6	8	47.9 ± 6.2	8	52.0 ± 3.3	8	42.4 ± 8.5	3	29.3 ± 5.0*
End of RP	4	53.8 ± 3.2	4	46.1 ± 5.3	4	52.8 ± 2.8	4	42.5 ± 6.1*	1	40.0
**TP (g/L)**										
The 1^st^ detect in AP	8	55.4 ± 3.3	8	55.5 ± 3.0	8	54.9 ± 3.7	8	53.6 ± 2.2	8	52.6 ± 3.5
The 2^nd^ detect in AP	8	57.0 ± 3.0	8	56.0 ± 3.3	8	56.5 ± 4.6	8	54.9 ± 2.8	8	55.1 ± 3.3
Day-3 (Before DD)	8	57.9 ± 3.6	8	54.9 ± 2.1	8	55.8 ± 2.9	8	55.5 ± 2.3	8	51.8 ± 3.2*
Day-14 (Before DD)	8	59.0 ± 3.9	8	56.8 ± 2.9	8	57.2 ± 3.7	8	56.3 ± 2.4	7	49.7 ± 2.8*
Day-27 (Before DD)	8	57.4 ± 4.9	8	55.0 ± 1.8	8	55.3 ± 3.7	8	54.7 ± 4.5	3	42.7 ± 3.1*
End of RP	4	58.3 ± 3.6	4	54.8 ± 1.6	4	55.9 ± 6.3	4	59.0 ± 12.9	1	52.9
**ALB(g/L)**										
The 1^st^ detect in AP	8	33.3 ± 2.5	8	35.2 ± 1.4	8	33.4 ± 1.8	8	33.7 ± 2.4	8	33.3 ± 2.1
The 2^nd^ detect in AP	8	34.0 ± 2.5	8	35.4 ± 1.7	8	33.5 ± 1.5	8	33.1 ± 2.4	8	33.6 ± 2.1
Day-3 (Before DD)	8	35.9 ± 2.3	8	35.3 ± 1.6	8	34.8 ± 1.8	8	34.2 ± 1.5	8	32.6 ± 2.5*
Day-14 (Before DD)	8	34.4 ± 1.9	8	33.8 ± 1.4	8	32.9 ± 1.8	8	31.8 ± 2.1*	7	26.7 ± 2.2*
Day-27 (Before DD)	8	34.2 ± 3.8	8	35.2 ± 1.2	8	34.0 ± 2.2	8	29.4 ± 3.4*	3	20.4 ± 0.6*
End of RP	4	35.7 ± 1.1	4	32.6 ± 2.4	4	35.8 ± 1.8	4	31.1 ± 3.4*	1	23.7
**A/G**										
The 1^st^ detect in AP	8	1.52 ± 0.15	8	1.77 ± 0.29	8	1.59 ± 0.28	8	1.71 ± 0.29	8	1.75 ± 0.28
The 2^nd^ detect in AP	8	1.50 ± 0.24	8	1.75 ± 0.29	8	1.51 ± 0.32	8	1.53 ± 0.21	8	1.58 ± 0.16
Day-3 (Before DD)	8	1.64 ± 0.15	8	1.82 ± 0.24	8	1.70 ± 0.27	8	1.65 ± 0.32	8	1.72 ± 0.25
Day-14 (Before DD)	8	1.40 ± 0.09	8	1.48 ± 0.10	8	1.39 ± 0.28	8	1.31 ± 0.17	7	1.17 ± 0.19
Day-27 (Before DD)	8	1.53 ± 0.35	8	1.78 ± 0.12	8	1.64 ± 0.30	8	1.21 ± 0.31	3	0.93 ± 0.11*
End of RP	4	1.64 ± 0.40	4	1.49 ± 0.21	4	1.85 ± 0.44	4	1.34 ± 0.74	1	0.81
**GLU (mmol/L)**										
The 1^st^ detect in AP	8	5.59 ± 0.40	8	5.46 ± 0.36	8	5.11 ± 0.37	8	5.44 ± 0.47	8	5.84 ± 0.48
The 2^nd^ detect in AP	8	5.35 ± 0.43	8	5.62 ± 0.16	8	5.23 ± 0.34	8	5.45 ± 0.38	8	5.58 ± 0.28
Day-3 (Before DD)	8	5.21 ± 0.37	8	5.51 ± 0.24	8	5.51 ± 0.30	8	5.83 ± 0.24*	8	6.21 ± 0.55*
Day-14 (Before DD)	8	6.03 ± 0.41	8	5.99 ± 0.21	8	5.88 ± 0.30	8	6.19 ± 0.34	7	5.77 ± 0.55
Day-27 (Before DD)	8	5.24 ± 0.36	8	4.97 ± 0.34	8	5.29 ± 0.23	8	5.13 ± 0.54	3	5.09 ± 1.06
End of RP	4	5.58 ± 0.44	4	5.42 ± 0.81	4	5.47 ± 0.78	4	5.53 ± 0.37	1	4.60
**TBIL (タµmol/L)**										
The 1^st^ detect in AP	8	1.0 ± 0.3	8	0.7 ± 0.3	8	0.7 ± 0.2	8	0.7 ± 0.2	8	0.8 ± 0.4
The 2^nd^ detect in AP	8	1.0 ± 0.3	8	0.9 ± 0.2	8	1.0 ± 0.3	8	1.1 ± 0.2	8	1.0 ± 0.3
Day-3 (Before DD)	8	1.3 ± 0.3	8	0.8 ± 0.4 *	8	1.1 ± 0.4	8	1.0 ± 0.3	8	0.8 ± 0.1*
Day-14 (Before DD)	8	0.9 ± 0.3	8	1.1 ± 0.3	8	1.0 ± 0.1	8	0.9 ± 0.5	7	38.2 ± 78.6
Day-27 (Before DD)	8	1.2 ± 0.4	8	0.9 ± 0.2 *	8	0.8 ± 0.2 *	8	4.1 ± 8.6	3	39.1 ± 46.6*
End of RP	4	0.9 ± 0.1	4	0.9 ± 0.2	4	0.8 ± 0.2	4	2.0 ± 2.3	1	7.6
**CHOL (mmol/L)**										
The 1^st^ detect in AP	8	4.24 ± 0.24	8	4.22 ± 0.25	8	4.47 ± 0.77	8	3.74 ± 0.43*	8	3.87 ± 0.48
The 2^nd^ detect in AP	8	4.18 ± 0.51	8	4.18 ± 0.54	8	4.54 ± 1.07	8	3.86 ± 0.46	8	3.89 ± 0.50
Day-3 (Before DD)	8	4.84 ± 0.49	8	4.41 ± 0.57	8	4.81 ± 0.60	8	4.13 ± 0.31*	8	4.79 ± 0.56
Day-14 (Before DD)	8	4.44 ± 0.43	8	4.59 ± 0.75	8	4.98 ± 0.70	8	3.81 ± 0.67	7	2.72 ± 1.16*
Day-27 (Before DD)	8	4.41 ± 0.33	8	4.41 ± 0.63	8	4.72 ± 1.12	8	2.86 ± 1.30*	3	0.88 ± 0.58*
End of RP	4	4.86 ± 0.69	4	4.03 ± 1.01	4	4.75 ± 0.44	4	3.50 ± 0.44	1	1.98
**TG (mmol/L)**										
The 1^st^ detect in AP	8	0.50 ± 0.11	8	0.43 ± 0.07	8	0.52 ± 0.09	8	0.51 ± 0.11	8	0.46 ± 0.10
The 2^nd^ detect in AP	8	0.53 ± 0.09	8	0.53 ± 0.10	8	0.56 ± 0.07	8	0.56 ± 0.08	8	0.52 ± 0.13
Day-3 (Before DD)	8	0.41 ± 0.06	8	0.41 ± 0.07	8	0.40 ± 0.06	8	0.39 ± 0.06	8	0.37 ± 0.07
Day-14 (Before DD)	8	0.46 ± 0.07	8	0.45 ± 0.07	8	0.45 ± 0.07	8	0.43 ± 0.10	7	0.45 ± 0.18
Day-27 (Before DD)	8	0.41 ± 0.07	8	0.43 ± 0.07	8	0.45 ± 0.07	8	0.37 ± 0.08	3	0.39 ± 0.14
End of RP	4	0.36 ± 0.08	4	0.38 ± 0.04	4	0.40 ± 0.09	4	0.48 ± 0.10	1	0.40
**K** **^+^** **(mmol/L)**										
The 1^st^ detect in AP	8	5.22 ± 0.29	8	5.28 ± 0.50	8	5.02 ± 0.29	8	5.19 ± 0.20	8	5.25 ± 0.28
The 2^nd^ detect in AP	8	5.21 ± 0.25	8	5.05 ± 0.31	8	5.16 ± 0.34	8	5.27 ± 0.34	8	5.13 ± 0.26
Day-3 (Before DD)	8	4.80 ± 0.23	8	4.55 ± 0.27	8	4.50 ± 0.22 *	8	4.52 ± 0.20	8	4.16 ± 0.21*
Day-14 (Before DD)	8	4.87 ± 0.22	8	4.59 ± 0.30	8	4.59 ± 0.24 *	8	4.70 ± 0.16	7	4.01 ± 0.60*
Day-27 (Before DD)	8	4.68 ± 0.23	8	4.52 ± 0.29	8	4.59 ± 0.20	8	4.40 ± 0.24	3	4.34 ± 0.40
End of RP	4	4.82 ± 0.22	4	4.41 ± 0.18 *	4	4.82 ± 0.14	4	4.86 ± 0.22	1	4.74
**Na** **^+^** **(mmol/L)**										
The 1^st^ detect in AP	8	147 ± 1	8	148 ± 2	8	147 ± 2	8	146 ± 2	8	148 ± 2
The 2^nd^ detect in AP	8	144 ± 2	8	145 ± 2	8	145 ± 1	8	145 ± 2	8	145 ± 1
Day-3 (Before DD)	8	146 ± 1	8	145 ± 1	8	144 ± 1	8	145 ± 1	8	145 ± 2
Day-14 (Before DD)	8	147 ± 2	8	148 ± 2	8	147 ± 1	8	149 ± 2	7	148 ± 2
Day-27 (Before DD)	8	148 ± 3	8	147 ± 2	8	147 ± 1	8	147 ± 1	3	148 ± 1
End of RP	4	145 ± 3	4	145 ± 1	4	147 ± 2	4	146 ± 1	1	141
**Cl** **^-^** **(mmol/L)**										
The 1^st^ detect in AP	8	110.1 ± 1.4	8	110.3 ± 1.7	8	109.3 ± 1.5	8	109.1 ± 2.2	8	111.1 ± 2.8
The 2^nd^ detect in AP	8	108.2 ± 2.0	8	109.0 ± 2.1	8	109.2 ± 1.1	8	109.6 ± 1.3	8	109.7 ± 1.0
Day-3 (Before DD)	8	109.0 ± 1.5	8	109.3 ± 0.8	8	109.6 ± 0.9	8	111.2 ± 1.2*	8	111.5 ± 2.0*
Day-14 (Before DD)	8	110.3 ± 1.3	8	110.6 ± 1.2	8	110.1 ± 1.0	8	112.4 ± 1.2*	7	111.3 ± 3.2
Day-27 (Before DD)	8	110.7 ± 3.0	8	110.0 ± 1.3	8	110.5 ± 1.1	8	111.2 ± 1.9	3	117.1 ± 2.4*
End of RP	4	109.2 ± 3.8	4	108.9 ± 1.5	4	111.2 ± 2.6	4	112.2 ± 1.5	1	108.9
**Ca (mmol/L)**										
The 1^st^ detect in AP	8	1.73 ± 0.24	8	1.75 ± 0.17	8	1.87 ± 0.14	8	1.53 ± 0.21	8	1.68 ± 0.18
The 2^nd^ detect in AP	8	2.59 ± 0.12	8	2.71 ± 0.12	8	2.68 ± 0.09	8	2.51 ± 0.15	8	2.50 ± 0.10
Day-3 (Before DD)	8	3.01 ± 0.08	8	3.01 ± 0.08	8	2.97 ± 0.04	8	2.94 ± 0.07	8	2.82 ± 0.10*
Day-14 (Before DD)	8	2.98 ± 0.11	8	3.00 ± 0.12	8	2.94 ± 0.11	8	2.90 ± 0.07	7	2.72 ± 0.17*
Day-27 (Before DD)	8	2.90 ± 0.12	8	2.98 ± 0.07	8	3.00 ± 0.13	8	2.84 ± 0.18	3	2.45 ± 0.05*
End of RP	4	3.03 ± 0.19	4	2.94 ± 0.13	4	3.14 ± 0.19	4	3.03 ± 0.25	1	2.56
**P (mmol/L)**										
The 1^st^ detect in AP	8	1.53 ± 0.15	8	1.53 ± 0.06	8	1.47 ± 0.09	8	1.40 ± 0.17	8	1.50 ± 0.30
The 2^nd^ detect in AP	8	2.16 ± 0.13	8	2.13 ± 0.20	8	2.12 ± 0.11	8	2.14 ± 0.17	8	2.15 ± 0.20
Day-3 (Before DD)	8	2.10 ± 0.11	8	2.01 ± 0.19	8	1.85 ± 0.17	8	2.01 ± 0.22	8	1.87 ± 0.22
Day-14 (Before DD)	8	2.25 ± 0.12	8	2.21 ± 0.24	8	2.03 ± 0.19	8	2.09 ± 0.21	7	1.95 ± 0.14*
Day-27 (Before DD)	8	1.87 ± 0.16	8	1.86 ± 0.14	8	1.86 ± 0.16	8	1.92 ± 0.20	3	1.63 ± 0.21
End of RP	4	2.17 ± 0.14	4	1.95 ± 0.21	4	1.98 ± 0.27	4	1.98 ± 0.40	1	1.73
**AMY-P (U/L)**										
The 1^st^ detect in AP	8	238.7 ± 86.5	8	282.9 ± 58.8	8	254.0 ± 52.6	8	216.2 ± 61.5	8	192.8 ± 46.4
The 2^nd^ detect in AP	8	357.0 ± 97.2	8	428.2 ± 102.3	8	360.7 ± 59.4	8	336.7 ± 82.7	8	309.5 ± 62.5
Day-3 (Before DD)	8	390.4 ± 120.3	8	467.5 ± 81.8	8	378.9 ± 56.1	8	379.3 ± 90.2	8	255.7 ± 27.7*
Day-14 (Before DD)	8	416.9 ± 108.2	8	515.8 ± 89.0	8	416.6 ± 61.2	8	396.0 ± 87.7	7	377.2 ± 110.2
Day-27 (Before DD)	8	449.8 ± 147.8	8	550.6 ± 83.9	8	449.1 ± 53.3	8	455.5 ± 122.2	3	467.4 ± 68.0
End of RP	4	559.5 ± 100.5	4	512.7 ± 37.7	4	432.8 ± 41.2	4	458.7 ± 125.6	1	438.7
**LIPC (U/L)**										
The 1^st^ detect in AP	8	25.0 ± 7.3	8	31.8 ± 6.8	8	29.9 ± 11.6	8	25.1 ± 13.6	8	34.7 ± 12.5
The 2^nd^ detect in AP	8	31.8 ± 8.3	8	46.4 ± 15.7	8	36.8 ± 11.9	8	36.4 ± 16.7	8	42.8 ± 14.4
Day-3 (Before DD)	8	38.8 ± 17.7	8	49.3 ± 22.8	8	44.0 ± 17.4	8	37.7 ± 17.7	8	39.7 ± 13.2
Day-14 (Before DD)	8	41.9 ± 22.7	8	42.2 ± 11.2	8	40.5 ± 16.2	8	38.4 ± 17.7	7	72.1 ± 47.5
Day-27 (Before DD)	8	53.0 ± 34.5	8	57.3 ± 33.0	8	52.3 ± 38.5	8	50.5 ± 34.0	3	146.0 ± 67.6*
End of RP	4	70.2 ± 48.8	4	51.0 ± 9.6	4	49.6 ± 17.1	4	35.5 ± 24.2	1	51.9

AP, adaptation period; DD, drug delivery; RP, recovery period; ALP, alkaline phosphatase; ALT, alanine aminotransferase ; AST, glutamic-acetic transaminase; GGT, *γ*-glutamyl transpeptidase; CK, creatinine kinase; CK-MB, MB isoenzyme of creatine kinase; LDH, L-lactate dehydrogenase; Urea, ureum; Crea, creatinine; TP, total protein; ALB, albumin; A/G, albumin/globulin; Glu, glucose; TBIL, total bilirubin; CHOL, cholesterol; TG, triglyceride.

^1)^ Arctigenin 60 mg/kg group, four dogs (5F04, 5F02, 5F03, and 5M01) successively died, resulting in animal sample reduction.

*Compared with control group, P ≤ 0.05.

### Urine and Faces Examination

Compared with the control group at the same time point, arctigenin administration resulted in series of changes as follows: 1) On day 13, BLO was significantly increased in both the arctigenin 20 and 60 mg/kg treated groups (*p* < 0.5). 2) On day 29, GLU, BIL, and URO were increased in the 60 mg/kg treated groups (*p* < 0.5). Otherwise, all urine parameters were no different, either at a different period or at a different dosage (**Table 9**).

**Table 9:** The urine parameters of arctigenin (6, 20, and 60 mg/kg) administration by subcutaneous injection in dogs during drug exposure period (n = 8, 4 female, 4 male) and recovery period (n = 4, 2 female, 2 male per treatment group; results were presented as number).

Paremeters	Time/grade	Control	PEG-treatment	Arctigenin		
				6 mg/kg^1)^	20 mg/kg	60 mg/kg^2)^
**Color**		**Yellow/Brown (n/n)**				
	First of AP	8/0	8/0	8/0	8/0	8/0
	Second of AP	7/1	8/0	8/0	8/0	8/0
	Day 13 of DDP	8/0	8/0	8/0	8/0	6/1
	Day 29 of DDP	8/0	8/0	7/0	8/0	3/0
	End of RP	4/0	4/0	4/0	4/0	1/0
**Clarity**		**Clear/Turbid (n/n)**				
	First of AP	8/0	8/0	8/0	8/0	8/0
	Second of AP	8/0	8/0	8/0	8/0	8/0
	Day 13 of DDP	8/0	8/0	8/0	8/0	6/1
	Day 29 of DDP	8/0	8/0	7/0	8/0	3/0
	End of RP	4/0	4/0	4/0	4/0	1/0
**Glucose**		**0.0/5.5 (nmol/L, n/n)**				
	First of AP	8	8	8	8	8
	Second of AP	8	8	8	8	8
	Day 13 of DDP	8	8	8	8	7
	Day 29 of DDP*	8	8	7	7/1	3/0
	End of RP	4	4	4	4	1
**BIL**		**Negative/Slight/Moderate/Severe (n/n)**				
	First of AP	8/0/0/0	8/0/0/0	8/0/0/0	6/2/0/0	8/0/0/0
	Second of AP	7/0/0/1	8/0/0/0	8/0/0/0	8/0/0/0	8/0/0/0
	Day 13 of DDP	7/1/0/0	8/0/0/0	8/0/0/0	8/0/0/0	3/2/1/1
	Day 29 of DDP*	6/2/0	8/0/0/0	7/0/0/0	4/2/0/2	0/0/0/3
	End of RP	3/1/0/0	3/1/0/0	3/1/0/0	1/2/1/0	0/0/1/0
**KET**		**0.0/1.5 (mmol/L, n/n)**				
	First of AP	8/0	8/0	8/0	8/0	8/0
	Second of AP	8/0	8/0	8/0	8/0	8/0
	Day 13 of DDP	8/0	8/0	8/0	7/1	6/1
	Day 29 of DDP	8/0	8/0	7/0	8/0	2/1
	End of RP	4/0	4/0	4/0	2/2	0/1
**S.G**		**1.010/1.015/1.020/1.025/1.030**				
	First of AP	0/3/4/0/1	1/4/2/1/0	1/5/1/1/0	0/6/0/1/1	0/5/2/0/1
	Second of AP*	0/8/0/0/0	4/4/0/0/0	0/6/1/1/0	2/5/1/0/0	0/6/2/0/0
	Day 13 of DDP	1/5/2/0/0	1/6/1/0/0	2/5/1/0/0	1/5/1/1/0	2/2/2/0/1
	Day 29 of DDP	0/3/3/1/1	1/5/1/0/1	1/3/2/1/0	2/3/2/0/1	0/1/2/0/0
	End of RP	0/2/2/0/0	0/2/2/0/0	2/0/1/1/0	1/0/3/0/0	0/0/1/0/0
**Occult blood**		0/25/80/200 (Ery/uL, n/n)				
	First of AP	8/0/0/0	8/0/0/0	8/0/0/0	8/0/0/0	7/1/0/0
	Second of AP	7/0/0/1	8/0/0/0	8/0/0/0	8/0/0/0	7/1/0/0
	Day 13 of DDP*	8/0/0/0	7/1/0/0	8/0/0/0	4/1/3/0	2/0/1/4
	Day 29 of DDP	8/0/0/0	6/1/0/1	4/1/1/1	7/0/1/0	3/0/0/0
	End of RP	4/0/0/0	4/0/0/0	4/0/0/0	4/0/0/0	1/0/0/0
**PH**		**5.0/5.5/6.0/6.5/7.0/7.5/8.0/8.5/9.0 (n/n)**				
	First of AP	0/1/4/2/0/1/0	0/1/4/0/1/2/0	2/0/1/1/1/3/0	0/1/1/1/0/4/1	1/1/1/0/0/5/0
	Second of AP	0/0/0/1/0/7/0	0/0/0/0/1/3/4	0/0/1/0/0/7/0	0/0/0/0/1/4/3	0/0/0/0/0/6/2
	Day 13 of DDP	0/0/0/0/1/2/1/3/1	0/0/0/1/1/0/0/4/2	0/0/1/0/0/0/1/5/1	0/1/0/0/0/1/0/3/3	1/0/1/1/2/0/0/1/1
	Day 29 of DDP	0/1/0/0/5/2/0/0/0	0/1/1/0/1/0/0/5/0	0/1/0/0/3/0/1/1/1	0/1/1/0/0/1/0/3/2	0/0/1/0/1/0/0/1/0
	End of RP	0/0/0/0/2/1/0/1/0	0/0/0/0/1/2/0/1/0	0/0/0/2/0/1/1/0/0	0/0/0/0/2/2/0/0/0	0/0/0/0/0/1/0/0/0
**PRO**		**0.0/0.3/1.0/3.0 (g/L, n/n)**				
	First of AP	7/1/0/0	7/1/0/0	8/0/0/0	6/1/0/0	6/2/0/0
	Second of AP	6/1/0/1	4/0/4/0	4/4/0/0/	3/3/2/0	5/3/0/0
	Day 13 of DDP	4/2/2/0	4/4/0/0/	1/3/4/0	3/4/1/0	4/2/1/0
	Day 29 of DDP	5/1/2/0	6/2/0/0	5/1/1/0	3/4/1/0	0/2/1/0
	End of RP	1/2/1/0	3/0/1/0	0/4/0/0/	1/1/2/0	0/1/0/0
**URO.**		**3.2/16.0/33.0 (µmol/L, n/n)**				
	First of AP	8/0/0	8/0/0	8/0/0	7/1/0	8/0/0
	Second of AP	7/1/0	8/0/0	8/0/0	8/0/0	8/0/0
	Day 13 of DDP	8/0/0	8/0/0	8/0/0	8/0/0	5/2/0
	Day 29 of DDP*	8/0/0	8/0/0	7/0/0	6/1/1	1/1/1
	End of RP	4/0/0	4/0/0	4/0/0	3/1/0	0/1/0
**NIT.**		**Negative/Positive (n/n)**				
	First of AP	8/0	7/1	8/0	7/1	7/1
	Second of AP	8/0	7/1	8/0	8/0	8/0
	Day 13 of DDP	8/0	8/0	8/0	8/0	7/0
	Day 29 of DDP	8/0	8/0	7/0	8/0	3/0
	End of RP	4/0	4/0	4/0	4/0	1/0
**WBC**		**0/15/70/125/500 (Leu/µl, n/n)**				
	First of AP	2/0/4/2/0	3/3/2/0/0/	1/2/4/1/0	4/0/4/0/0	4/0/2/2/0
	Second of AP	4/0/3/1/0	4/1/2/0/1	1/0/7/0/0/	2/2/4/0/0/	1/3/1/3/0
	Day 13 of DDP	4/1/2/1/0	4/0/2/2/0	2/1/3/1/1	2/2/3/1/0	2/0/3/0/2
	Day 29 of DDP	5/0/2/1/0	5/0/0/2/1	3/1/2/0/1	6/1/0/1/0	3/0/0/0/0
	End of RP	4/0/0/0/0	4/0/0/0/0	4/0/0/0/0	4/0/0/0/0	1/0/0/0/0

BIL, bilirubin; URO, urobilinogen; KET, ketones; PRO, protein; NIT, nitrite; GLU, glucose; S.G, specific gravity; LEU, leucocytes;

^1)^ One animal’s urine (3F02) in arctigenin-6 mg/kg group has not been collected on day 29^th^; the total number was 7 (n = 7).

^2)^ Arctigenin 60 mg/kg group, four dogs (5F04, 5F02, 5F03, and 5M01) successively died, resulting in animal sample reduction.

*Compared with control group, P ≤ 0.05.

The feces of all animals were studied during the drug exposure and recovery periods. The color, parasites, RBC, WBC, epithelial cells, fat granules, and BLO showed no differences among all groups (**Table 10**).

**Table 10:** The feces parameters of arctigenin (6, 20, and 60 mg/kg) administration by subcutaneous injection in dogs during drug exposure period (n = 8, 4 female, 4 male) and recovery period (n = 4, 2 female, 2 male per treatment group; results were presented as number).

Parameters	Time grade	Control	PEG treatment	Arctigenin treatment		
				6 mg/kg	20 mg/kg	60 mg/kg^1)^
**Color**		Yellow (n)				
	First of AP	8	8	8	8	8
	Second of AP	8	8	8	8	8
	Day 13 of DDP	8	8	8	8	6
	Day 29 of DDP	8	8	8	8	3
	End of RP	4	4	4	4	1
**Character**		Soft/Loose stools (n/n)				
	First of AP	8	8	8	8	8
	Second of AP	8	8	8	8	8
	Day 13 of DDP	8/0	8/0	8/0	8/0	5/1
	Day 29 of DDP	8	8	8	8	3
	End of RP	4	4	4	4	1
**Parasite**		Negative (n)				
	First of AP	8	8	8	8	8
	Second of AP	8	8	8	8	8
	Day 13 of DDP	8	8	8	8	6
	Day 29 of DDP	8	8	8	8	3
	End of RP	4	4	4	4	1
**RBC**		Negative (n)				
	First of AP	8	8	8	8	8
	Second of AP	8	8	8	8	8
	Day 13 of DDP	8	8	8	8	6
	Day 29 of DDP	8	8	8	8	3
	End of RP	4	4	4	4	1
**WBC**		Negative/Positive (n/n)				
	First of AP	8/0	8/0	8/0	8/0	8/0
	Second of AP	7/1	8/0	8/0	8/0	8/0
	Day 13 of DDP	8/0	8/0	8/0	8/0	6/0
	Day 29 of DDP	8/0	8/0	8/0	7/1	3/0
	End of RP	4/0	4/0	4/0	4/0	1/0
**Epithelium**		Negative/Positive (n/n)				
	First of AP	8/0	8/0	7/1	8/0	8/0
	Second of AP	8/0	8/0	8/0	8/0	8/0
	Day 13 of DDP	7/1	8/0	8/0	8/0	6/0
	Day 29 of DDP	8/0	8/0	8/0	8/0	3/0
	End of RP	4/0	4/0	4/0	4/0	1/0
**Fat**		Negative (n)				
	First of AP	8	8	8	8	8
	Second of AP	8	8	8	8	8
	Day 13 of DDP	8	8	8	8	6
	Day 29 of DDP	8	8	8	8	3
	End of RP	4	4	4	4	1

^1)^ Arctigenin 60 mg/kg group, four dogs (5F04, 5F02, 5F03, and 5M01) successively died, resulting in animal sample reduction.

### ctn-T and Bone Marrow Examination

Compared with the control group at the same time point (drug exposure and recovery period), neither PEG nor arctigenin treatment resulted in a cTn-T index change (data not shown).

At the end of the recovery period, the percentage of other cells in the PEG-treated group was elevated (*p* < 0.05). In addition, compared with the control group, the parameters of bone marrow smears were no different among all the animals (**Table 11**).

**Table 11:** The cTn-T and bone marrow examination of arctigenin (6, 20, and 60 mg/kg) administration by subcutaneous injection in dogs during drug exposure period (n = 8, 4 female, 4 male) and recovery period (n = 4, 2 female, 2 male per treatment group; results were presented as mean ± S.D.).

Parameters Time	Control		PEG Treatment		Arctigenin-6 mg/kg		Arctigenin-20 mg/kg		Arctigenin-60 mg/kg^1)^	
	End of DE	End of RP	End of DE	End of RP	End of DE	End of RP	End of DE	End of RP	End of DE	End of RP
**Myeloid erythroid ratio**	1.85 ± 0.31	1.94 ± 0.57	1.89 ± 0.15	1.78 ± 0.14	1.75 ± 0.21	1.67 ± 0.12	1.95 ± 0.43	1.53 ± 0.37	1.06 ± 1.43	0.87
**Granulocyte (%)**	60.1 ± 4.4	59.4 ± 5.7	59.4 ± 2.4	59.4 ± 1.5	58.9 ± 1.8	57.3 ± 2.4	60.1 ± 4.8	55.1 ± 5.5	50.5 ± 53.5	43.5
**Myeloblast (%)**	0.0 ± 0.0	0.6 ± 0.3	0.3 ± 0.3	0.5 ± 0.4	0.0 ± 0.0	0.5 ± 0.4	0.1 ± 0.3	0.6 ± 0.8	1.0 ± 0.0	0.5
**Progranulocyte (%)**	0.6 ± 0.5	1.0 ± 0.9	0.5 ± 0.4	1.1 ± 0.6	1.0 ± 0.4	0.5 ± 0.4	1.1 ± 0.5	0.9 ± 0.9	0.5 ± 1.5	0.5
**Neutrophil (%)**	56.5 ± 3.9	54.0 ± 7.4	54.0 ± 3.1	54.0 ± 2.2	54.8 ± 1.3	52.8 ± 2.4	56.4 ± 4.7	51.5 ± 5.9	45.5 ± 51.0	40.0
**Eosinophils (%)**	3.0 ± 2.0	3.8 ± 1.4	4.6 ± 1.7	3.8 ± 1.7	3.1 ± 1.2	3.5 ± 0.9	2.5 ± 1.1	2.1 ± 1.1	3.5 ± 1.0	2.5
**Basophils (%)**	0.0 ± 0.0	0.0 ± 0.0	0.0 ± 0.0	0.0 ± 0.0	0.0 ± 0.0	0.0 ± 0.0	0.0 ± 0.0	0.0 ± 0.0	0.0 ± 0.0	0.0
**RBC (%)**	33.0 ± 3.0	32.1 ± 6.5	31.5 ± 1.5	33.5 ± 1.9	33.9 ± 2.9	34.3 ± 1.0	31.6 ± 5.2	37.4 ± 7.1	47.5 ± 37.5	50.0
**Pronormoblasts (%)**	0.8 ± 0.5	0.3 ± 0.3	0.1 ± 0.3	0.5 ± 0.4	0.1 ± 0.3	0.1 ± 0.3	0.4 ± 0.3	0.4 ± 0.5	0.0 ± 0.0	0.5
**Prorubricyte (%)**	2.1 ± 1.0	2.4 ± 0.9	2.5 ± 0.4	2.0 ± 0.7	1.3 ± 0.5	1.4 ± 0.9	1.4 ± 0.8	2.1 ± 1.3	1.5 3.5	1.5
**Rubricyte (%)**	16.3 ± 1.3	15.4 ± 5.2	16.0 ± 1.6	18.5 ± 3.2	17.6 ± 4.1	17.3 ± 2.4	16.8 ± 3.8	20.8 ± 1.7	27.5 ± 18.0	28.5
**Metarubricyte (%)**	13.9 ± 2.8	14.1 ± 2.1	12.9 ± 0.6	12.5 ± 0.7	14.9 ± 2.3	15.5 ± 2.0	13.1 ± 2.1	14.1 ± 5.3	18.5 16.0	19.5
**Lymphocyte (%)**	5.6 ± 1.7	7.5 ± 1.3	7.3 ± 1.4	5.0 ± 0.7	5.6 ± 1.3	7.8 ± 1.8	5.4 ± 2.9	5.8 ± 2.8	2.0 6.0	5.0
**Monocyte (%)**	0.0 ± 0.0	0.1 ± 0.3	0.1 ± 0.3	0.1 ± 0.3	0.4 ± 0.5	0.1 ± 0.3	0.1 ± 0.3	0.1 ± 0.3	0.0 0.0	0.0
**Other cells (%)**	1.3 ± 1.3	0.9 ± 0.5	1.8 ± 0.6	2.0 ± 0.7 *	1.3 ± 1.3	0.6 ± 0.5	2.8 ± 1.2	1.6 ± 0.6	0.0 3.0	1.5
**Megalokaryocyte (n)**	104 ± 45	116 ± 40	140 ± 32	114 ± 31	80 ± 39	90 ± 18	106 ± 69	102 ± 9	72 66	105

^1)^ Arctigenin 60 mg/kg group, four dogs (5F04, 5F02, 5F03, and 5M01) successively died, resulting in animal sample reduction.

*Compared with control group, P ≤ 0.05.

### Viscera Weights and Viscera/Body Weights Index

Compared with the control group, neither the PEG group nor the arctigenin-treated group had changes in viscera weights and viscera/body weights of heart, liver, spleen, lung, kidney, brain, adrenal glands, ovary, uterus, testis, and epididymis (*p* > 0.05). However, at the end of the drug exposure period, one dog’s kidney weight and kidney/body weight index were both increased by arctigenin 20 mg/kg treatment (4M02). In the arctigenin 60 mg/kg treatment group, viscera weights and viscera/body weights of the spleen (5F01) and kidney (5F01, 5M03) were increased at the end of drug exposure, and all animals’ ovary, uterus, testis, and epididymis weights were decreased after the recovery period (**Table 12**).

**Table 12:** Viscera weights and viscera/body weights index of arctigenin (6, 20, and 60 mg/kg) administration by i.h in beagle dogs at the end of drug delivery (n = 4, 2 male and 2 female) and recovery period (n = 4, 2 female, 2 male per treatment group; results were presented as mean ± S.D.).

Parameters Time	Control		PEG treatment		Arctigenin treatment					
					6 mg/kg		20 mg/kg		60 mg/kg^1)^	
	End of DE	End of RP	End of DE	End of RP	End of DE	End of RP	End of DE	End of RP	End of DE	End of RP
**Viscera weights (kg)**										
Body weight	8.68 ± 0.35	10.00 ± 0.45	9.21 ± 0.29	9.79 ± 0.61	9.20 ± 0.50	9.69 ± 0.34	8.76 ± 0.27	9.36 ± 1.11	7.20 ± 10.30	8.45
Brain	75.93 ± 11.31	74.83 ± 5.98	78.36 ± 4.00	68.84 ± 3.14	77.52 ± 5.75	77.81 ± 6.05	72.95 ± 4.84	74.59 ± 5.04	76.04 78.91	77.57
Heart	73.19 ± 4.15	80.18 ± 6.75	67.92 ± 10.12	78.60 ± 6.29	72.75 ± 5.38	75.89 ± 6.16	72.83 ± 7.56	71.61 ± 3.25	63.59 76.41	76.83
Liver	257.34 ± 19.04	237.72 ± 9.92	274.03 ± 34.12	226.56 ± 14.60	290.00 ± 16.98	247.53 ± 6.08	277.88 ± 28.58	224.92 ± 16.82	255.57 ± 330.77	256.91
Spleen	20.15 ± 2.45	25.59 ± 1.67	24.90 ± 4.30	26.99 ± 3.84	25.29 ± 4.98	26.35 ± 3.58	23.21 ± 1.22	26.17 ± 3.24	73.14 28.56	47.75
Lung	83.82 ± 6.80	87.62 ± 6.10	75.78 ± 5.72	87.49 ± 7.01	85.99 ± 7.43	90.55 ± 13.26	76.69 ± 6.65	86.51 ± 3.19	64.61 74.26	90.48
Thymus	9.40 ± 4.34	15.34 ± 7.30	10.49 ± 3.04	11.08 ± 1.02	7.77 ± 1.92	13.03 ± 4.20	6.51 ± 3.06	8.59 ± 5.43	3.21 6.71	4.67
Kidney	44.46 ± 7.21	45.34 ± 2.83	43.55 ± 3.86	45.84 ± 3.11	41.57 ± 3.40	43.73 ± 8.17	52.57 ± 14.57	48.33 ± 3.22	52.64 ± 63.44	50.90
Adrenalin	0.93 ± 0.13	1.11 ± 0.26	0.98 ± 0.14	0.92 ± 0.17	0.94 ± 0.17	1.07 ± 0.12	1.08 ± 0.18	1.02 ± 0.12	0.93 ± 1.44	1.14
Ovary	0.57 ± 0.70	0.67 ± 0.76	0.65 ± 0.63	0.69 ± 0.53	0.45 ± 0.89	0.79 ± 0.73	0.62 ± 0.72	1.10 ± 0.60	0.47	NA
Uterus	2.94 ± 1.76	1.32 ± 2.00	3.24 ± 0.60	2.78 ± 3.57	3.56 ± 2.64	3.18 ± 3.19	1.75 ± 1.30	15.77 ± 1.75	0.71	NA
Testis	11.95 ± 13.66	11.16 14.16	8.45 ± 10.01	10.45 ± 8.63	11.52 ± 11.08	14.01 ± 14.34	5.18 ± 5.06	6.41 ± 7.36	6.67	6.92
Epididymis	2.27 ± 2.53	3.66 ± 2.76	2.81 ± 2.50	2.39 ± 2.15	2.92 ± 2.06	2.51 ± 4.71	1.27 ± 1.66	1.39 ± 1.72	1.75	1.37
**Viscera/Body weight index (g/100 g)**										
Brain	0.878 ± 0.149	0.750 ± 0.071	0.851 ± 0.057	0.707 ± 0.074	0.845 ± 0.083	0.803 ± 0.061	0.832 ± 0.038	0.809 ± 0.143	1.056 0.766	0.918
Heart	0.845 ± 0.068	0.805 ± 0.103	0.739 ± 0.126	0.804 ± 0.055	0.791 ± 0.052	0.783 ± 0.059	0.830 ± 0.069	0.775 ± 0.116	0.883 0.742	0.909
Liver	2.976 ± 0.320	2.378 ± 0.052	2.970 ± 0.293	2.317 ± 0.139	3.153 ± 0.090	2.556 ± 0.070	3.167 ± 0.244	2.442 ± 0.477	3.550 3.211	3.040
Spleen	0.233 ± 0.033	0.256 ± 0.021	0.271 ± 0.053	0.278 ± 0.051	0.275 ± 0.055	0.272 ± 0.033	0.265 ± 0.015	0.285 ± 0.069	1.016 0.277	0.565
Lung	0.969 ± 0.103	0.876 ± 0.045	0.823 ± 0.059	0.895 ± 0.070	0.935 ± 0.063	0.933 ± 0.113	0.874 ± 0.052	0.932 ± 0.098	0.897 0.721	1.071
Thymus	0.107 ± 0.046	0.155 ± 0.080	0.113 ± 0.031	0.114 ± 0.014	0.084 ± 0.018	0.134 ± 0.043	0.074 ± 0.035	0.087 ± 0.053	0.045 0.065	0.055
Kidney	0.515 ± 0.100	0.454 ± 0.027	0.473 ± 0.037	0.470 ± 0.048	0.452 ± 0.038	0.450 ± 0.070	0.598 ± 0.159	0.522 ± 0.079	0.731 ± 0.616	0.602
Adrenalin	0.011 ± 0.002	0.011 ± 0.002	0.011 ± 0.002	0.009 ± 0.002	0.010 ± 0.002	0.011 ± 0.001	0.012 ± 0.002	0.011 ± 0.002	0.012 ± 0.014	0.014
Ovary	0.006 ± 0.008	0.007 ± 0.007	0.007 ± 0.007	0.007 ± 0.006	0.005 ± 0.010	0.008 ± 0.007	0.007 ± 0.008	0.012 ± 0.006	0.0065	NA
Uterus	0.032 ± 0.021	0.014 ± 0.019	0.036 ± 0.007	0.030 ± 0.039	0.041 ± 0.029	0.033 ± 0.033	0.021 ± 0.015	0.168 ± 0.018	0.010	NA
Testis	0.140 ± 0.161	0.114 ± 0.140	0.091 ± 0.105	0.099 ± 0.086	0.121 ± 0.114	0.151 ± 0.142	0.057 ± 0.057	0.082 ± 0.070	0.0648	0.082
Epididymis	0.027 ± 0.030	0.037 ± 0.027	0.030 ± 0.026	0.023 ± 0.022	0.031 ± 0.021	0.027 ± 0.047	0.014 ± 0.019	0.018 ± 0.016	0.017	0.016

^1)^Arctigenin 60mg/kg group, 4 dogs (5F04, 5F02, 5F03, and 5M01) successively death result in animal samples reduction.

### Toxicological Study

To determine its toxicokinetic characteristics, the plasma concentration–time profiles of arctigenin were detected following subcutaneous administration of 6, 20, and 60 mg/kg arctigenin in beagle dogs (**Figure S3**), and the TK parameters are shown in **Table S1**. After drug administration, the average values of T_max_ were 75 to 195 min and 67.5 to 240 min after the first and the last drug exposure by subcutaneous injections, respectively (**Table S1**).

After the first drug exposure, the ratios of female and male plasma AUC_0-t_ were 1:3.2: 8 and 1:2.7:5.9 when animals underwent different dosage administration (6, 20, and 60 mg/kg ratio was 1:3.3:10). In addition, these ratios were changed to 1:3.8:11.8 and 1:4.9:12.3 after the last drug exposure, respectively. AUC_last_ showed no difference compared with AUC_first_ in both male and female dogs, and the ratio of AUC_last_/AUC_first_ was 0.9:1.9, which suggested that there was no drug accumulation (**Figure S4**).

### Pathological Examination

Gross anatomy. 1) After drug exposure, injection sites were observed as dark red in all animals (including PEG- and arctigenin-treated animals). In two dogs in the PEG group (2/4), one dog in the arctigenin 6 mg/kg group (1/4), one dog in the arctigenin 20 mg/kg group (1/4), and one dog in the arctigenin 6 mg/kg group (1/2), volume enlargement of the subaxillary lymph node was observed. Further, arctigenin treatment (both 20 and 60 mg/kg) resulted in testicular and epididymal volume reduction. Arctigenin 60 mg/kg injection led to liver and spleen volume enlargement, uterine volume reduction, and pancreatic swelling. 2) After the recovery period, dark red was still observed subcutaneously at the injection site in the PEG group (four dogs, 4/4), the arctigenin 6 mg/kg group (three dogs, 3/4), the arctigenin 20 mg/kg group (four dogs, 4/4), and the arctigenin 60 mg/kg group (one dog, 1/1). Furthermore, 60 mg/kg arctigenin administration resulted in a reduction in the dogs’ thymus volume.

**Figure 3:**
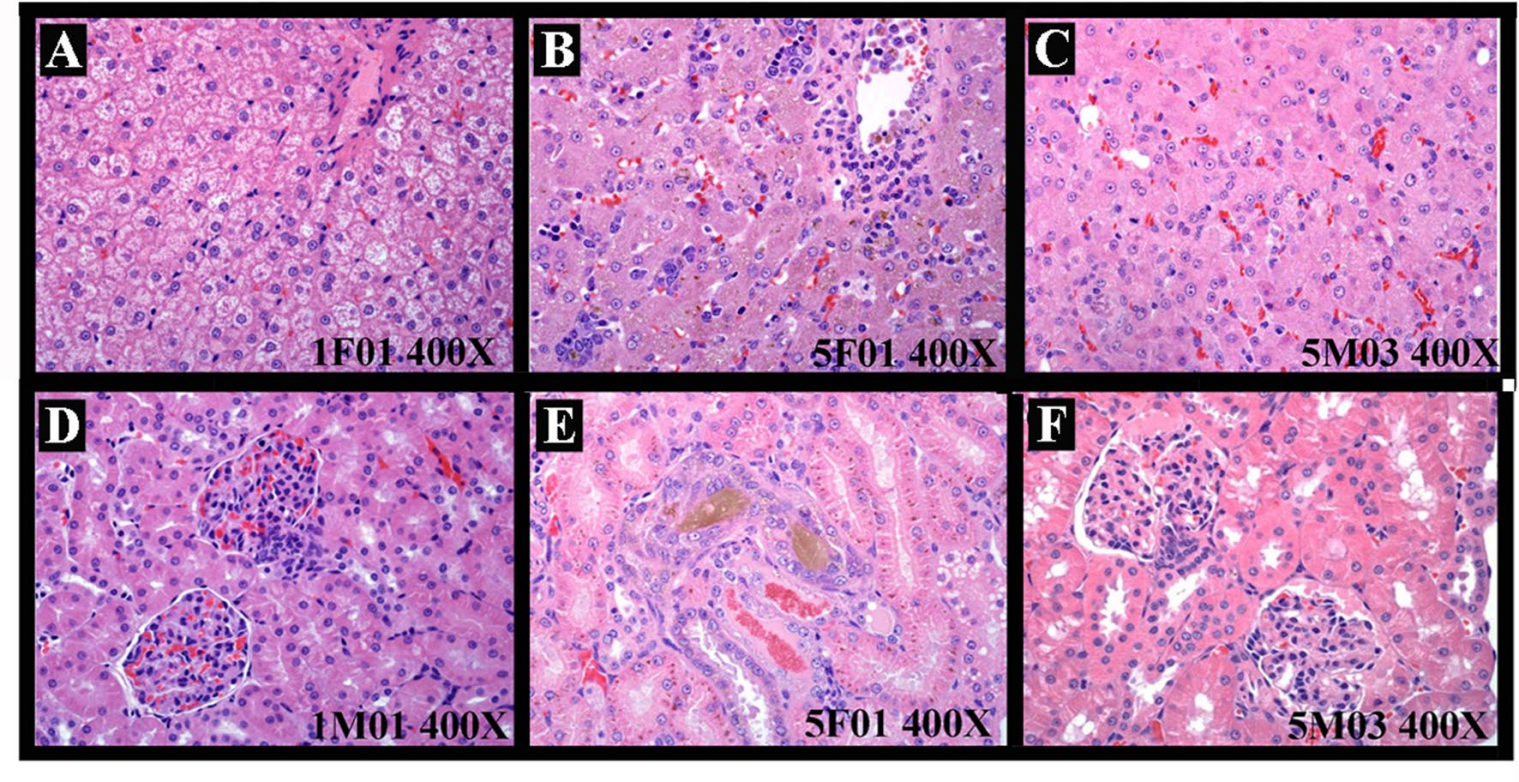
The representative histopathological changes of animals resulted from test articles after drug exposure. Hepatocyte degeneration/necrosis, cholestasis, and inflammatory cell infiltration (**A**, control group. **B**, arctigenin 60 mg/kg group) and extramedullary hematopoiesis and increased nuclear mitosis (**C**, arctigenin 60 mg/kg group). Renal tubular basophilic degeneration/regeneration, pigmentation, and hyaline droplets in (**D**, control group; **E**, **F**, arctigenin 60 mg/kg group).

Histopathology Examination. 1) After drug exposure, slight-severe, moderate-severe, moderate-severe, and severe-serious test articles deposition/inflammation were observed in the PEG group (four dogs, 4/4), the arctigenin 6 mg/kg group (four dogs, 4/4), the arctigenin 20 mg/kg group (four dogs, 4/4), and the arctigenin 60 mg/kg group (two dogs, 2/2), respectively. Compared with the control group, the primary pathological changes of drug exposure sites were drug subcutaneous deposition, foreign body giant cell responses, extensive edema, bleeding, necrosis, and inflammatory cell infiltration. In addition, in the arctigenin 60 mg/kg treatment group, a foreign body response was observed in one dog’s subcutaneous tissue of the mammary gland, which may have resulted from the spread of the series of pathological changes at the drug exposure site. In the lymphoid hematopoietic system, test article subcutaneous deposition at the subaxillary lymph node was observed in the PEG group (slight, two dogs, 2/4), the arctigenin 6 mg/kg group (slight, two dogs, 2/4), the arctigenin 20 mg/kg group (slight-moderate, three dogs, 3/4), and the arctigenin 60 mg/kg group (slight-moderate, two dogs, 2/2). In addition, slight-severe red cells increased/phagocytosed in the sinus. In the thymus, arctigenin 20 and 60 mg/kg administration resulted in slight-severe thymus atrophy. In the spleen, arctigenin 60 mg/kg administration caused severe EMH, slight foam-like macrophage aggregation, and white pulp atrophy. In the digestive system, hepatocyte degeneration/necrosis, cholestasis, and inflammatory cell infiltration were observed in both the arctigenin 20 and 60 mg/kg group (**Figure 3A, B**). In addition, arctigenin 60 mg/kg exposure resulted in EMH and increased nuclear mitosis (**Figure 3C**). Furthermore, both arctigenin 20 and 60 mg/kg treatment caused slight mucous epithelial hyperplasia of the gallbladder, and slight pancreas acinus hypertrophy combined with interstitial edema resulted from arctigenin 60 mg/kg administration. In the urinary system, moderate renal tubular basophilic degeneration/regeneration, pigmentation, and hyaline droplets in the Bowman capsule of the renal tubules were observed in the arctigenin 60 mg/kg group (**Figure 3D–F**). Besides that, slight EMH of the adrenal glands was caused by arctigenin 60 mg/kg administration. 2) After the recovery period, the pathological changes of drug exposure sites in all treatment animals (including PEG- and arctigenin-treated groups) were observed including drug subcutaneous deposition, foreign body giant cell responses, and extensive fibroplastic proliferation, which were significantly ameliorated in PEG, arctigenin 6, and arctigenin 20 mg/kg treatment groups after the 14-day recovery, but not in the arctigenin 60 mg/kg treated animals. In accordance with the injection sites, slight-moderate test articles deposition and pigmentation of the subaxillary lymph node were observed in the arctigenin administration groups (including 6, 20, and 60 mg/kg). Arctigenin 20 mg/kg treatment resulted in submaxillary lymph node deposition/foreign body reaction. In the spleen, even after recovery, slight and severe EMH and foam-like macrophage aggregation still existed in the PEG group, the arctigenin 20 mg/kg group, and the arctigenin 60 mg/kg group. Although thymus atrophy was improved in the control and PEG treated groups, severe and slight thymus atrophy remained in the arctigenin 20 and 60 mg/kg groups. In addition, slight-moderate cholestasis, slight hepatocyte degeneration/necrosis, inflammatory cell infiltration, and EMH were observed in both the arctigenin 20 and 60 mg/kg treated groups.

## Discussion

Herbalism is the study and use of plants intended for cure or as diet supplements. Herbs have been used through much of human history, and are still widely used today, especially in China, Korea, and Japan, and even in western countries (Maegawa et al., 2014; Bae et al., 2015; Izzo et al., 2016; Fleischer et al., 2017; Jia et al., 2017). Modern medicine recognizes herbalism as alternative and pseudoscience medicine because of its dependence on multicomponents and empiricism, but not based on evidence gathered by scientific methods (2007). Modern medicines use many plant-extracted or -derived compounds such as opium, aspirin, and quinine (Yanai et al., 2016). The World Health Organization (WHO) states that approximately 25% of modern drugs in the United States are derived from plants, and worldwide at least 7000 drugs are plant derivatives (Fabricant and Farnsworth, 2001).

In all Chinese medicine, the topic of toxicity of certain substances has been described from the earliest records regarding their use. Since Traditional Chinese Medicine (TCM) has been more and more popular in western countries, the concern about the potential toxicities of TCM is increasing simultaneously because the efficacy and toxicity testing are based on traditional knowledge, but not on laboratory evidence (Shaw, 2010). The potential toxicities in some cases could be confirmed by research. For example, aristolochic acid, contained in the genus *Aristolochia*, has been reported to result in kidney toxicity associated with kidney failure and the development of cancer (Ernst, 1998; Gold and Slone, 2003). The herbs indicated as being hepatotoxic include horse chestnut, pennyroyal, and pyrrolizidine alkaloids (Elvin-Lewis, 2001; 2010). In 2012, the WHO appealed to formulate relative policy and international standards for evaluating the safety and efficacy of traditional medicine (Ocvirk et al., 2013). The process of setting up safety evaluation criteria of herbal remedies, especially to evaluate the consequences of long-term use, is underway. Arctigenin, as a natural product macular, is extracted from Greater burdock. Greater burdock extracts contain several compounds, including flavonoids, lignans, tannins, phenolic acids, alkaloids, and terpenoids. Greater burdock is a plant used in TCM. Arctigenin is a lignin, belonging to monomer compound. We could evaluate its toxicity according to a regular method.

In this study, the subchronic toxicities (28-day consecutive drug exposure and 14-day recovery) of arctigenin in a series of concentrations (6, 20, and 60 mg/kg/day) were administered, and reversibility profiles, such as body weights, food consumption, and hematological, biochemical, histopathological, and toxicokinetic parameters, were evaluated in beagle dogs.

PEG-400 (polyethylene glycol 400), a low-molecular-weight grade of polyethylene glycol, is widely used in a variety of pharmaceutical formulations such as oral liquid and eye drops because of its clarity, colorless, solubilization, low toxicity, and well tolerance (Tber et al., 2015). Importantly, arctigenin could be completely dissolved in PEG400. In this study, both PEG- and arctigenin-treated groups were given high concentration (80%) and volume (1.2 ml/kg) of PEG-400 as a solvent for consecutive 28-day injections, which resulted in obvious stress reactions as follows. 1) During the drug administration, the symptoms such as growling, struggling, and vomiting were observed in dogs, and repeated biting by dogs of the injection sites resulted in trauma, ulceration, and scabbing. 2) Compared with PEG, arctigenin induced local hemolysis, and WBC increases were more significant, especially in the arctigenin 60 mg/kg group, and the subchronic administration resulted in an increase of RET%, a decrease in coagulation function (PLT and PT prolonged), and a decrease in ALB and A/G. 3) Varying degrees of deposition/inflammation of dogs’ subcutaneous reactions, foreign body reactions, and polycythemia of dogs’ lymph nodes were observed in PEG- and arctigenin-treated groups by histopathology examinations.

All these abnormal hematological, biochemical, and histopathological changes were associated with the dosage of arctigenin. However, after the 2-week recovery period, hematological and biochemical parameters were not abnormal, and pathological symptoms were relieved. The main toxic target organs of PEG and/or arctigenin include hematopoietic, digestive, and urinary systems. Effects of arctigenin on lymphatic hematopoietic system including WBC and PLT elevation, macrophage accumulation in spleen, thymus atrophy, and so on were caused by inflammation at the injection sites. In addition, toxicity of arctigenin on digestive system, e.g., liver enlargement, degeneration and necrosis, pancreatic cysts, and cholestasis, is the main reason of dogs’ death. Indicators of urine routine were significantly elevated and renal tubule was basophilic or regenerative after 60 mg/kg arctigenin treatment, which was related to a hemolytic reaction in the kidney. Arctigenin (60 mg/kg) also influenced dogs’ lung and heart. There inflammation happened after arctigenin treatment. These data supplied a reference for clinical trial and drug registration. Due to the limits of arctigenin’s poor oral bioavailability, subcutaneous injected administration was selected. Toxicity of arctigenin by oral administration after drug formulation change will be researched in the future.

## Conclusion

This study provides a systematic investigation of the toxic proﬁles of arctigenin *in vivo*. In summary, the no observed adverse effect level (NOAEL) of arctigenin was less than 6 mg/kg in dogs, which corresponded to human dose 3.21 mg/kg. This result provides a reference for the safety of arctigenin at a clinical trial. The main targets of arctigenin toxicities were heart, liver, and kidney. Hepatocyte necrosis, renal tubular degeneration, and cardiotoxicity are the leading reasons of death.

## Data Availability Statement

The raw data supporting the conclusions of this manuscript will be made available by the authors, without undue reservation, to any qualified researcher.

## Ethics Statement

The experimental protocols were approved by the Animal Ethics Committee of Lunan Pharmaceutical Group Co. Ltd. All animal experiments complied with the ARRIVE guidelines was carried out in accordance with the National Institutes of Health guide for the care and use of Laboratory animals (NIH Publications No. 8023, revised 1978).

## Author Contributions

Y-sR and J-cY contributed to the conception and design of the study and approved the final version to be submitted. Y-sR, Y-gL, and JL contributed to drafting the article. JL and Y-sR performed data analysis and interpretation. Y-JT and TP performed the histopathological examination. Y-gL, L-hP, and F-fY performed the blood and urine collection and detection. F-fY and TP performed the drug exposure, body weight, and food consumption detection. ZL, J-cY, and G-MZ performed the sample detection for toxicokinetics assays.

## Funding

This work was supported by the grants from the Shandong Province Science and Technology Major Project (grant no.2015ZDJQ05004) and National Science and Technology Support Program (grant no. 2012CB724001), gratefully received from the Generic Manufacture Technology of Chinese Traditional Medicine and Lunan Pharmaceutical Group Co. Ltd.

## Conflicts of Interest

JL, L-hP, F-fY, TP, Y-jT, G-MZ, ZL, J-cY, and Y-sR are employed by Lunan Pharmaceutical Group Co. Ltd.

The remaining author declares that the research was conducted in the absence of any commercial or financial relationships that could be construed as a potential conflict of interest.

## Abbreviations

RBC, red blood cell count; HGB, hemoglobin; HCT, hematocrit; MCV, mean corpuscular volume; MCH, mean corpuscular hemoglobin; MCHC, mean corpuscular hemoglobin concentration; RET%, reticulocyte (%); WBC, white blood cell count; NEU, neutrophil; LYM, lymphocyte; MONO, monocyte; EOS, eosinophil; BASO, basophil; PLT, platelet; PT, prothrombin time; and APTT, activated partial thromboplastin time. AST, aspartate aminotransferase; ALT, alanine aminotransferase; CPK, creatine phosphokinase; CK-MB, myocardial bound creatine kinase; ALP, alkaline phosphatase; LDH, L-lactate dehydrogenase; GGT, γ-glutamyltranspeptidase; urea, ureum; TP, total protein; ALB, albumin; A/G, albumin/globulin; GLU, blood glucose; TBIL, total bilirubin; crea, creatinine; CHOL, cholesterol; TG, triglyceride; AMY-P, amylopsin; LIPC, lipidase. GLU, glucose; BIL, bilirubin; KET, ketone; SG, specific gravity; BLO, occult blood; PRO, protein; URO, urobilinogen; NIT, nitrites; WBC, white blood cell.
