# Supplementary material for: Toxicity Study of 28-Day Subcutaneous Injection of Arctigenin in Beagle Dogs
Source: Front Pharmacol. 2019 Oct 16;10:1218. doi: 10.3389/fphar.2019.01218 (PMC6807677; doi:10.3389/fphar.2019.01218)
Supplement: Supplementary file 1 [file DataSheet_1.docx]

**
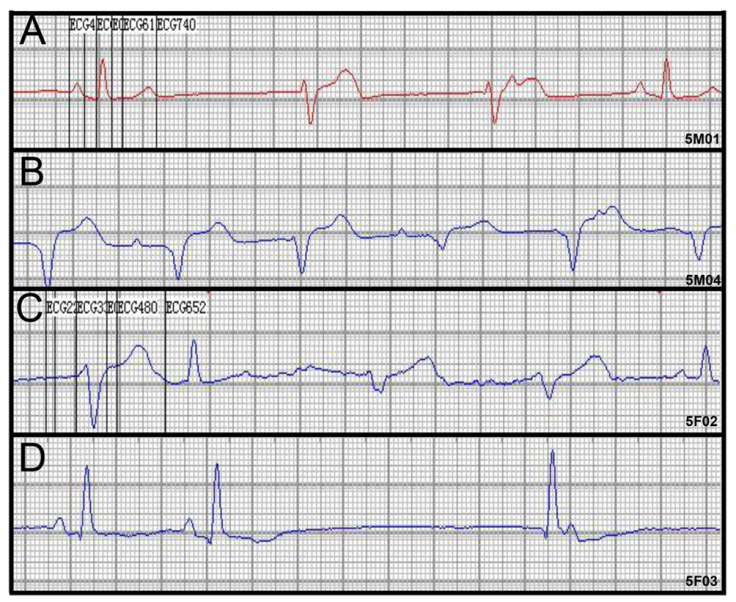
**

**Figure S1. The Typical Electrocardiogram Examination of Articulo Mortis Animals.**


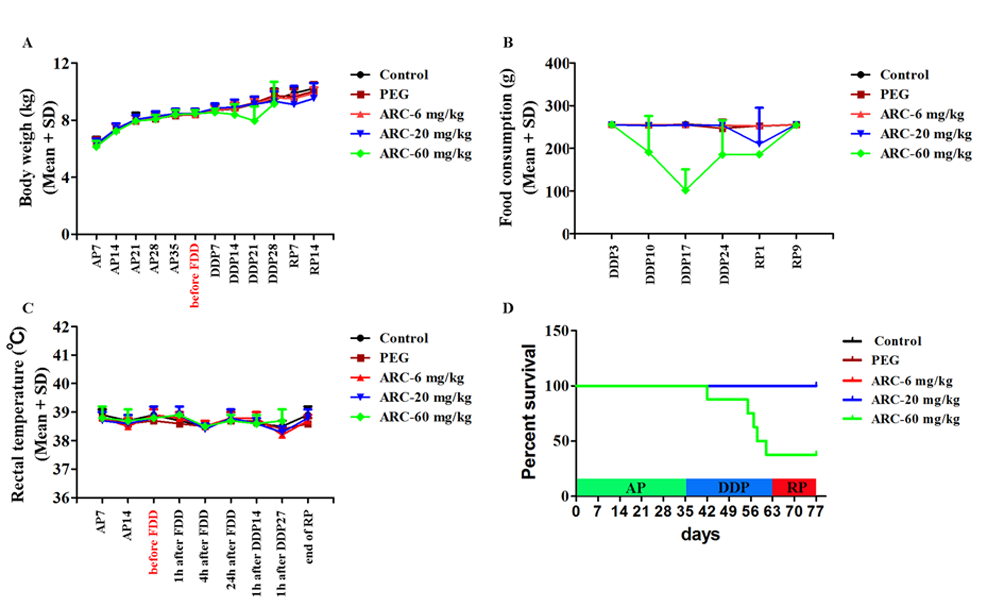


**Figure S2.** Body Weight (**A**), Food Consumption (**B**), Rectal Temperature (**C**), and Survial (**D**) of Beagle Dogs treated with *Arctigenin* at sub-chronic toxicity levels. (n=8 per treatment group; results are presented as the mean + S.D.). PEG=Polyethylene Glyeo, ARC=arctigenin, AP=adaptation, DDP=drug delivery period, RP=recovery period, FDD=first drug delivery.

**
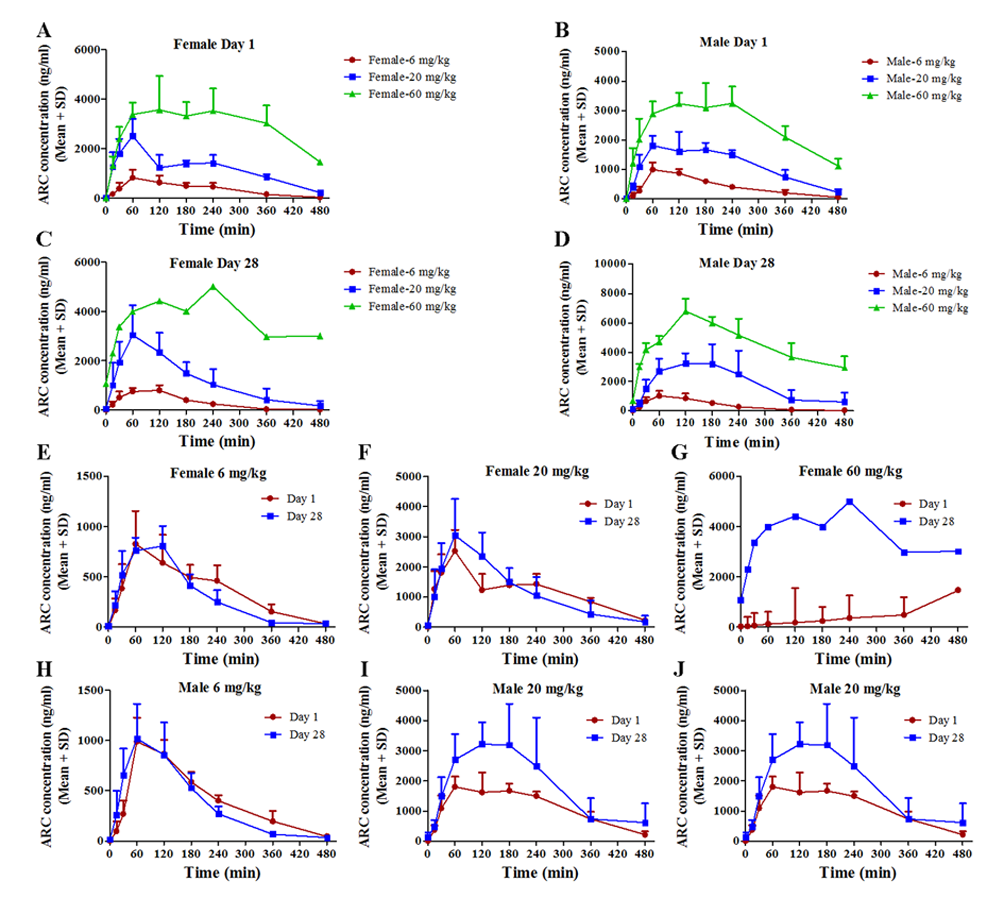
**

**Figure S3.** The plasma concentration vs. time profiles of Arctigenin in dogs after the first (Day 1, **A**, female. **B**, male) and last (Day 28, **C**, female, **D**, male) subcutaneous administrations by Arctigenin at different doses. The plasma concentrations of Arctigeninin dogs’ plasma after s.i Arctigenin at the dosage of 6 mg/kg/day (**E**, female, **H**, male), 20 mg/kg/day (**F**, female, **I**, male), and 60 mg/kg/day (**G**, female, **J**, male).

(Results are presented as the mean + SD). ARC, arctigenin.


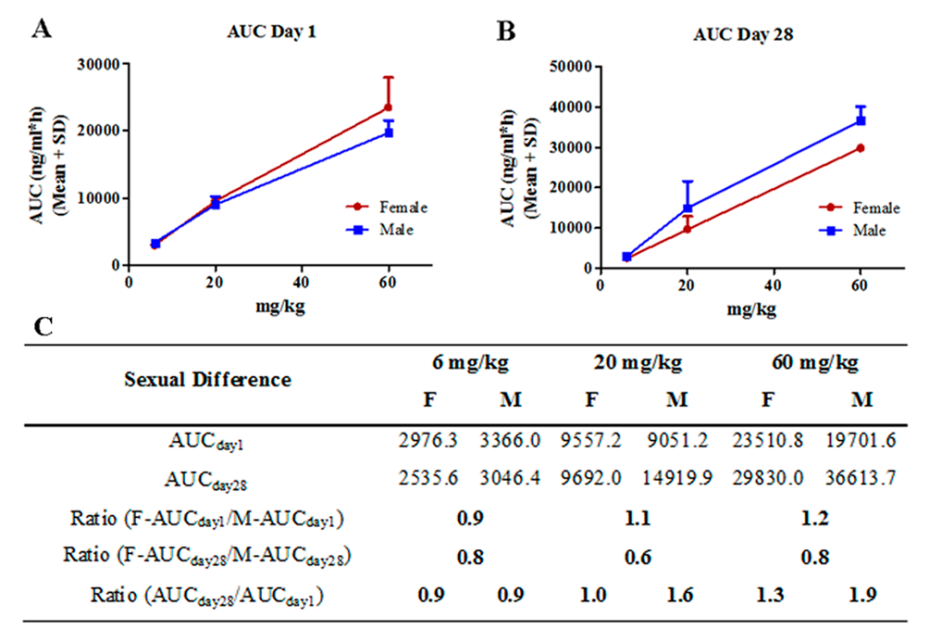


**Figure S4.** The exposed quantity of Arctigenin (6, 20, and 60 mg/kg) in dogs after the first (Day 1, **A**) and last (Day 28, **B**) subcutaneous administrations. (**C**) Relative exposed quantity and ratios between male and female dogs. Results are presented as the mean + SD). AUC, area under the curve, F-AUC, female area under the curve, M-AUC, male area under the curve.

**Table S1.Toxicokinetic parameters of Arctigenin (6-, 20, and 60 mg/kg) administration by subcutaneous injection in beagle dogs at the first drug exposure (day 1) and last drug exposure (day28) (results were presented as Mean & S.D.).**

|  | | | **Variable** | | | | | | | | |
| --- | --- | --- | --- | --- | --- | --- | --- | --- | --- | --- | --- |
|  |  |  | **AUC_last_ (min*ng/mL)** | | | **C_max_** | | | **T_max_** | | |
| **Group** | **Study day** | **Gender** | **n** | **Mean** | **SD** | **n** | **Mean** | **SD** | **n** | **Mean** | **SD** |
| 6mg/kg | 1 | Female | 4 | 2976.3 | 409.1 | 4 | 926.3 | 253.4 | 4 | 90.0 | 34.6 |
|  |  | Male | 4 | 3366.0 | 92.6 | 4 | 1019.0 | 212.1 | 4 | 75.0 | 30.0 |
|  | 28 | Female | 4 | 2535.6 | 605.7 | 4 | 839.5 | 147.1 | 4 | 105.0 | 30.0 |
|  |  | Male | 4 | 3046.4 | 680.0 | 4 | 1105.8 | 364.5 | 4 | 90.0 | 34.6 |
| 20mg/kg | 1 | Female | 4 | 9557.2 | 327.1 | 4 | 2550.0 | 663.6 | 4 | 105.0 | 90.0 |
|  |  | Male | 4 | 9051.2 | 1171.0 | 4 | 1952.5 | 368.5 | 4 | 120.0 | 49.0 |
|  | 28 | Female | 4 | 9692.0 | 3266.2 | 4 | 3245.0 | 1071.8 | 4 | 67.5 | 37.7 |
|  |  | Male | 4 | 14919.9 | 6693.1 | 4 | 3578.8 | 854.2 | 4 | 135.0 | 57.4 |
| 60mg/kg | 1 | Female | 4 | 23510.8 | 4403.8 | 4 | 3958.8 | 1071.3 | 4 | 195.0 | 133.0 |
|  |  | Male | 4 | 19701.6 | 1842.7 | 4 | 3518.8 | 364.8 | 4 | 120.0 | 69.3 |
|  | 28 | Female | 1 | 29830.0 | -- | 1 | 5000.0 | -- | 1 | 240.0 | -- |
|  |  | Male | 2 | 36613.7 | 3479.8 | 2 | 6830.0 | 777.8 | 2 | 150.0 | 42.4 |

On the day 28^th^, only one female and two male dogs in Arctigenin-60 mg/kg treatment group.
